# Two-Dimensional Electronic Spectroscopy Resolves Relative Excited-State Displacements

**DOI:** 10.1021/acs.jpclett.3c03420

**Published:** 2024-03-06

**Authors:** Giovanni Bressan, Dale Green, Garth A. Jones, Ismael A. Heisler, Stephen R. Meech

**Affiliations:** † School of Chemistry, Norwich Research Park, University of East Anglia, Norwich NR4 7TJ, United Kingdom; ‡ Instituto de Fisica, Universidade Federal do Rio Grande do Sul, 91509-900 Porto Alegre, RS, Brazil

## Abstract

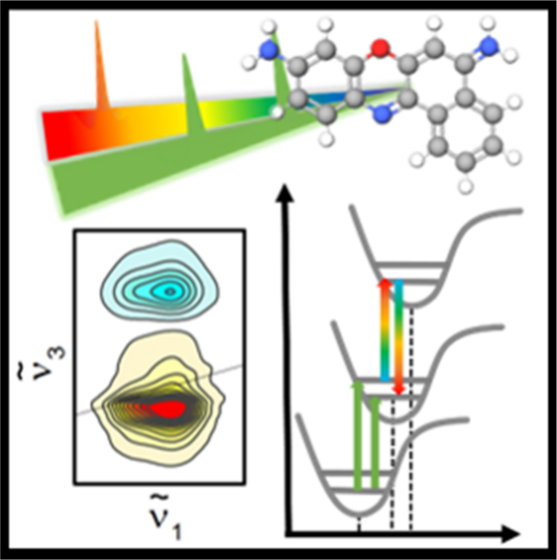

Knowledge of relative displacements between potential energy surfaces (PES) is critical in spectroscopy and photochemistry. Information on displacements is encoded in vibrational coherences. Here we apply ultrafast two-dimensional electronic spectroscopy in a pump–probe half-broadband (HB2DES) geometry to probe the ground- and excited-state potential landscapes of cresyl violet. 2D coherence maps reveal that while the coherence amplitude of the dominant 585 cm^–1^ Raman-active mode is mainly localized in the ground-state bleach and stimulated emission regions, a 338 cm^–1^ mode is enhanced in excited-state absorption. Modeling these data with a three-level displaced harmonic oscillator model using the hierarchical equation of motion-phase matching approach (HEOM-PMA) shows that the S_1_ ← S_0_ PES displacement is greater along the 585 cm^–1^ coordinate than the 338 cm^–1^ coordinate, while S_*n*_ ← S_1_ displacements are similar along both coordinates. HB2DES is thus a powerful tool for exploiting nuclear wavepackets to extract quantitative multidimensional, vibrational coordinate information across multiple PESs.

The role of coherent superpositions of vibrational, vibronic, and electronic states in fundamental photophysical, photochemical, and photobiological processes has been thoroughly investigated by ultrafast spectroscopies.^1−7^ In the past 20 years, two-dimensional electronic spectroscopy (2DES) emerged as the ideal tool for the investigation of such effects in condensed-phase systems due to its ability to decongest spectra and recover rephasing (photon-echo) and nonrephasing signals.^8−13^ Pump–probe 2DES is a four-wave mixing technique in which a pair of ultrashort time-ordered collinear pump pulses coherently excites electronic or vibronic transitions. Those are interrogated by a delayed, visible probe pulse which is, in the “half-broadband (HB)” design, a compressed white-light continuum (WLC).^14,15^ On top of population dynamics, the pump pair initiates nuclear wavepacket motion, detected as amplitude modulations during the waiting (population) time *T*, defined as the time interval between the second pump and the WLC probe pulses. The frequency, dephasing time, spectral position, and amplitude of such oscillatory features encode dynamical information on the ground- and excited-state potential energy surfaces (PESs).^16−20^ Importantly, the amplitude of a vibrational coherence reports on the displacement between the initial and final PESs along a specific normal mode coordinate.^21−25^

We performed HB2DES on the system cresyl violet perchlorate (CV) in ethanol (EtOH). CV is a cationic oxazine dye that has been widely studied by one-color 2DES, leading to a wealth of data on population and coherence dynamics associated with the ground-state bleach (GSB) and stimulated emission (SE) transitions.^26−30^ By addressing dynamics in this model system, we show that the extended range of the WLC probe in HB2DES enables access to higher electronic states, S_*n*_, via excited-state absorption (ESA), revealing coherent dynamics that are inaccessible in most one-color experiments. We further show that a detailed analysis of these ESA measurements allows the recovery of displacements among higher excited states through the relative amplitude of wavepackets arising from multiple Raman-active modes coupled to different electronic transitions. The ability to initiate coherent dynamics and probe the structure of higher-lying excited states is becoming increasingly important in ultrafast photochemistry. Several recent experiments show that the excitation of electronic states higher than S_1_ leads to novel photophysical and photochemical processes not accessible by other pathways, encompassing processes such as novel reactive channels in photochromics, charge injection for photovoltaics, and anti-Kasha emission.^31−35^ The present data suggest that coherence spectroscopies provide new information and potentially suggest routes to control such phenomena.

Experimental absorptive HB2DES spectra of CV in EtOH at waiting times of *T* = 100, 160, 500 fs are shown in Figure 1b–d. Steady-state absorption and emission (ν̃_*exc*_ = 17000 cm^–1^) of CV are shown in Figure 1a as violet solid and dotted lines, respectively, where the pump spectrum is also shown. Dashed lines indicate the frequency of the 0–0 S_1_ ← S_0_ transition, determined by the crossing point of the normalized steady-state absorption and emission spectra at 16250 cm^–1^. Calculated HB2DES spectra at *T* = 100, 160, and 500 fs are shown in Figure 1f–h, while the steady-state absorption and pump spectra used in the 2DES simulations are reported in Figure 1e, following the same color code as in Figure 1a. The spectra in Figure 1f–h were calculated using the hierarchical equations of motion (HEOM) method that fully accounts for solvent effects through multiple overdamped baths in combination with the equation of motion–phase-matching approach (EOM–PMA), explicitly accounting for the electric fields of the pump and probe pulses, as previously described by Green et al.^36^ Full details of the model are given in the SI.

**Figure 1:**
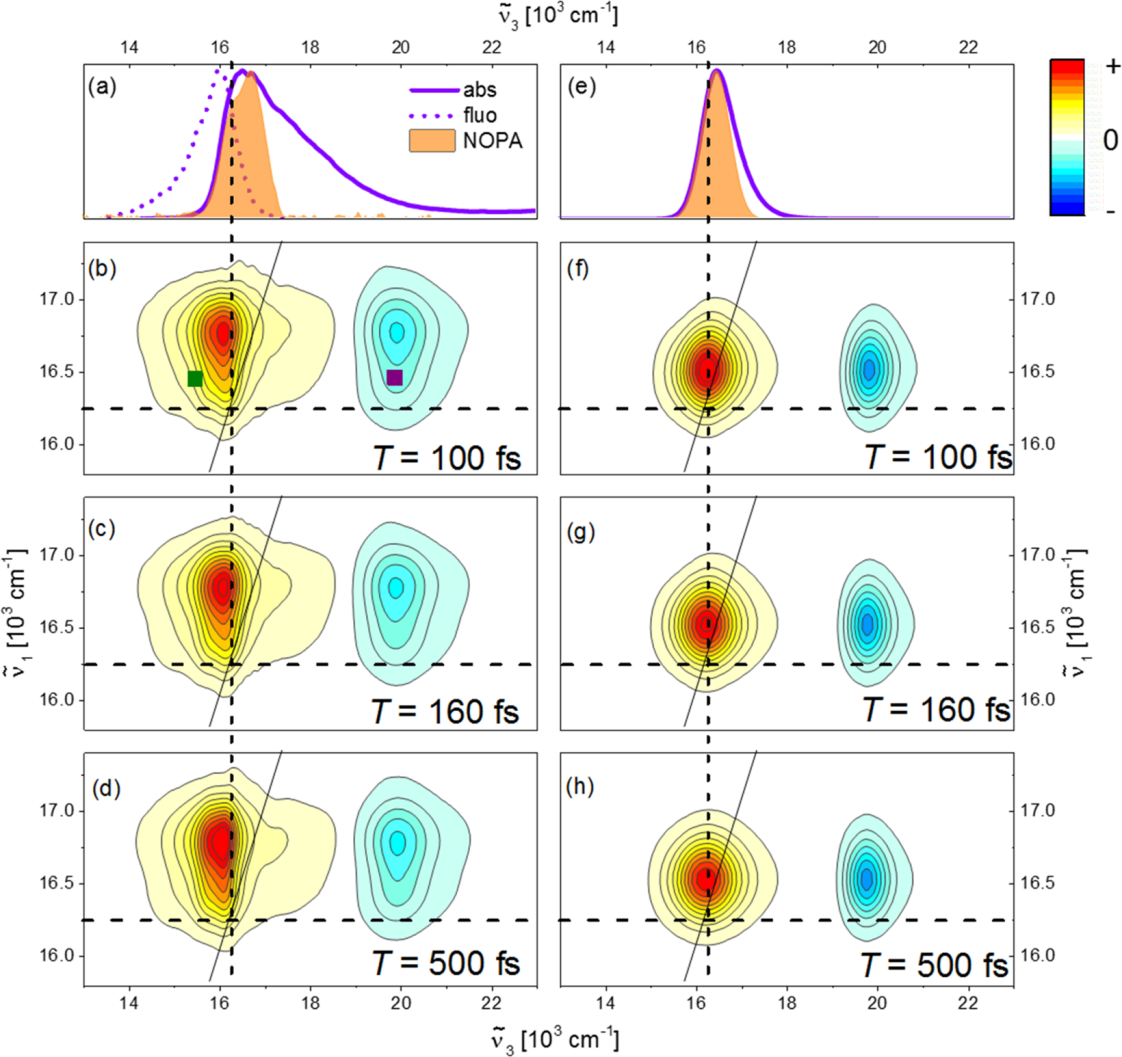
(a) Normalized steady-state experimental absorption (solid) and emission (dotted) of cresyl violet (CV) in ethanol. The NOPA pump spectrum used for the HB2DES measurements is shown as a shaded orange area. The experimental absorptive 2D spectra of CV at *T* = 100, 160, and 500 fs are shown in (b–d), respectively. The intensity is given by 21 contour lines; positive signals are shown in yellow-orange-red, and negative signals are shown in blue. All spectra are normalized to the 2DES positive amplitude at *T* = 100 fs. Dashed lines indicate the position of the 0–0 transition (16250 cm^–1^). (e) The same as (a) for the simulated 2DES of CV. (Details of the simulation are given below and in Supporting Information.) (f–h) Simulated absorptive 2DES spectra of CV at *T* = 100, 160, and 500 fs, respectively. Dark-green and purple squares indicate the coordinates at which the traces shown in Figure 2 are taken.

The experimental and modeled absorptive 2D spectra are in very good agreement in terms of both peak positioning and broadening, indicating that correct Hamiltonian and bath parameters (reported in the SI) are employed for the modeled spectra of CV in EtOH. The calculated steady-state absorption (to which the GSB is related) is narrower than the measured spectra because the model accounts for the broadening with only a single low-frequency vibration coupled to the electronic transition.

Absorptive 2DES spectra present a convoluted positive GSB and SE (GSB + SE) region, matching the steady-state absorption and emission spectra, for probe (ν̃_3_) wavenumbers between 14000 and 18800 cm^–1^ and a negative ESA band located in the ν̃_3_ range of 19000–21500 cm^–1^. These results are in agreement with literature 2DES and visible fs transient absorption (fsTA) of CV.^26−28,37−39^ 2D line shapes display negligible evolution over the waiting time *T* (1.2 ps), suggesting that any reshaping due to spectral diffusion occurs on a time scale comparable to the ∼50 fs instrument response function (IRF, shown in Figure S1). This is in agreement with the <50 fs 2DES spectral diffusion dynamics observed by Lu et al.^28^ for CV in methanol.

Traces extracted from the GSB + SE and ESA regions of the absorptive 2DES spectra as a function of time *T* are shown in the SI (Figure S2). We observed an ∼ps SE rise time (ν̃_1_ = 16500; ν̃_3_ = 15800 cm^–1^) previously reported in a 2DES study of CV by Carbery et al.^27^ Over the same waiting time window, the negative ESA region (ν̃_1_ = 16500; ν̃_3_ = 19900 cm^–1^) decays on a similar time scale. Such dynamics report on the evolution within the S_1_ PES and could reflect the intramolecular vibrational energy redistribution (IVR) or structural dynamics, whose characterization is beyond the scope of the present work.

The population relaxation as a function of *T* for the GSB + SE and ESA features is accompanied by strong oscillations due to coherent wavepacket dynamics. This is exemplified by the residuals of a global fit (two exponential terms, τ_1_ = 350 fs and τ_2_ = 5000 fs + offset) to the measured rephasing real response at two points in the 2DES, marked in Figure 1b by dark-green and purple squares (Figure 2).

**Figure 2:**
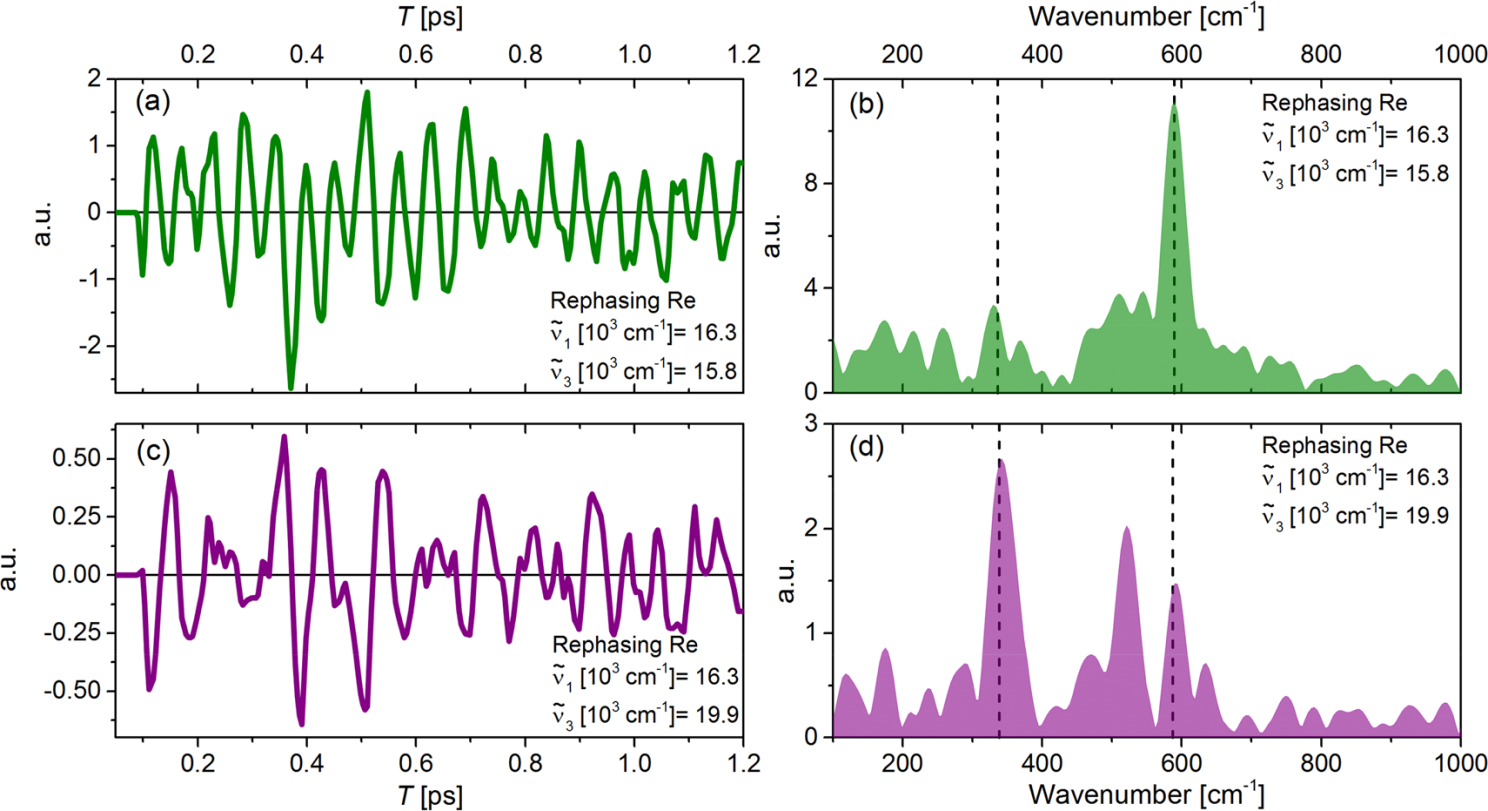
(a) Time-domain oscillatory residuals of the real part of the rephasing 2DES of cresyl violet at the coordinates marked by the green square in Figure 1b. (b) Fourier transform of the residuals in (a). (c) The same as (a), at the coordinate marked by the purple square in Figure 1b. (d) The same as (b) for the oscillatory residuals shown in (c).

Figure 2a shows the residuals at excitation and detection frequencies of ν̃_1_ = 16500 cm^–1^ and ν̃_3_ = 15800 cm^–1^ (GSB + SE region). The residuals show a single dominant frequency whose dephasing time outlasts the measured experimental waiting time (*T*) window of 1200 fs. After zero padding (to the second-closest power of 2) and Fourier transforming of the residuals in Figure 2a, the impulsive Raman spectrum obtained is shown in Figure 2b. This spectrum is dominated by a mode at 585 cm^–1^, which has been reported previously as a strongly allowed, Raman-active oxazine ring elongation mode.^21,28,40^ Weaker contributions at 338 and 520 cm^–1^ are also present, in agreement with previous 2DES, spontaneous resonance (RR), impulsive stimulated Raman (ISRS), and fsTA studies of CV.^21,28,38−40^ The real part of the rephasing residuals over *T* at ν̃_1_ = 16300 and ν̃_3_ = 19900 cm^–1^ in the ESA region is shown in Figure 2c. In contrast to the GSB + SE data in Figure 2a, multiple frequencies with comparable amplitudes are evident. The corresponding impulsive Raman spectrum is shown in Figure 2d. Its most intense feature is a 338 cm^–1^ mode, assigned by Vogel et al. to a skeletal deformation,^40^ which exceeds the intensity in the 585 cm^–1^ mode by 1.5 times. This mode is present in Figure 2b (GSB + SE region) but is ∼4 times weaker than the 585 cm^–1^ mode. Such a “reversal” of the 338 and 585 cm^–1^ relative amplitudes between the GSB + SE and ESA regions was previously reported in a femtosecond coherence spectroscopy (FCS) study of CV by Fitzpatrick et al.^38^

Deeper insights into the excitation and detection frequency dependence of nuclear wavepackets can be obtained by analyzing the coherence beat maps of the two well-resolved Raman-active modes at 338 and 585 cm^–1^. The objective is to resolve the origin of the striking (ν̃_1_; ν̃_3_) coordinate-dependent amplitude reversal in the “single trace” impulsive Raman spectra reported in Figure 2b,d. The positive and negative beat maps of the 338 and 585 cm^–1^ modes are obtained by stacking rephasing (or nonrephasing) real and imaginary 2D spectra as a function of waiting time *T*. These are then globally fit to a multiexponential decaying function to isolate and remove the “slow” (ps–ns) population dynamics. The residuals of the real and imaginary global fit are then summed as *R*_*Re*_ + *iR*_Im_ (where *R*_*Re*/Im_ are the real or imaginary residuals matrices) to yield a complex-valued matrix which is Fourier transformed over *T*. Finally, beat maps of specific Raman-active modes are obtained by slicing along ν̃_*T*_ (i.e., the frequency dimension obtained by Fourier transforming over *T*), the 3D data set at the wavenumber of the Raman-active modes of interest.^13,41,42^ A schematic illustration of this procedure is shown in the Supporting Information, Figure S3. Experimental and calculated rephasing ±338/585 cm^–1^ beat maps are shown in Figure 3, while the corresponding nonrephasing data are shown in the SI (Figure S6). Rephasing and nonrephasing measured and calculated beat maps are overlaid on contour lines indicating the experimental, or calculated, absorptive 2DES of CV at *T* = 500 fs, reproduced from Figure 1d,h.

**Figure 3:**
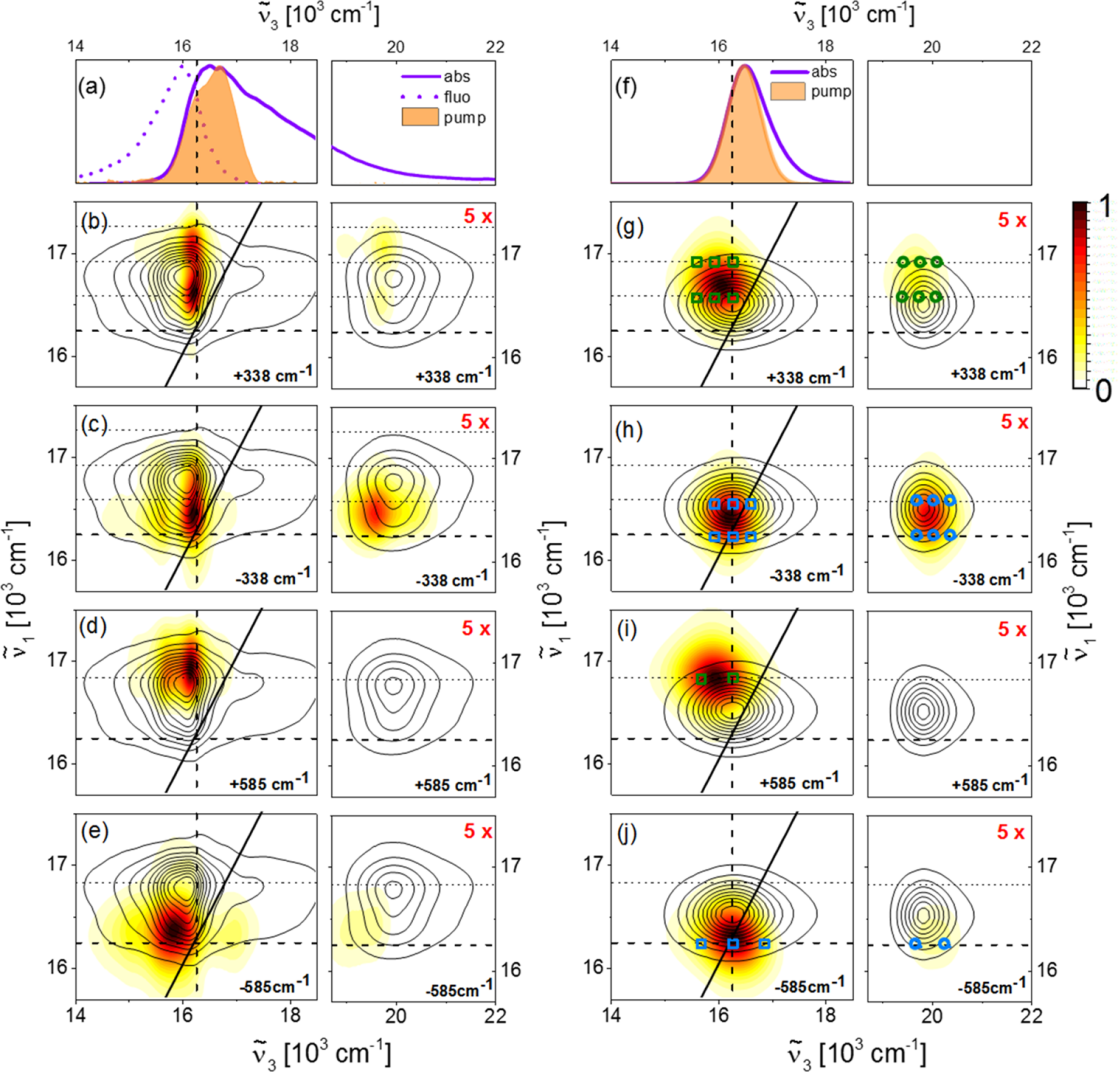
(a) Normalized steady-state absorption (solid) and emission (dashed) spectra of CV along with the NOPA pump spectrum. The probe axes are broken at 18400 cm^–1^ to highlight that the beat map amplitudes are rescaled by 5× in the ESA regions. (b, c) Rephasing positive and negative beat maps of the 338 cm^–1^ Raman-active mode shown as white-yellow-red heat maps and are all normalized to 1. (d, e) Same as in (b, c) for the 585 cm^–1^ Raman-active mode. Contour lines showing the real part of the experimental absorptive 2D spectrum (*T* = 500 fs, reproduced from Figure 1d) are overlaid in (b–e). Vertical and horizontal thick dashed lines are drawn at the 0–0 electronic transition frequency (16250 cm^–1^), and horizontal thin dashed lines are drawn at +1, 2 quanta of vibrational excitation (+3 for the 338 cm^–1^ beat maps). (f) Pump and CV steady-state absorption spectra used in the simulated 2DES of CV. (g, h) are the same as (b, c) and (i, j) are the same as (d, e) for the simulated rephasing positive and negative beat maps of the 338 and 585 cm^–1^ modes. Contour lines showing the real part of the calculated absorptive 2D spectrum (*T* = 500 fs, reproduced from Figure 1h) are overlaid in g–j. Green (blue) squares and circles indicate the locations predicted by positive (negative) SE and ESA double-sided Feynman diagrams contributing to the experimentally detected and calculated beat maps, respectively.

Figure 3 shows the excitation and detection wavenumber-resolved beat map amplitudes of the rephasing positive and negative coherences due to the 338 cm^–1^ (b and c respectively) and 585 cm^–1^ (d and e) modes compared to the steady-state absorption and emission spectra and pump spectrum (a). Figure 3g–j shows the ±338/ ± 585 cm^–1^ beat maps modeled using the steady-state absorption and pump spectra shown in Figure 3f. In Figure 3b–e and Figure 3g–j, the ESA region of the beat maps is multiplied by a factor of 5, enabling better visualization of the weaker signals detected in such a region.

All of the experimental beat maps present intense signals around the GSB + SE maximum (ν̃_3_ = 16000 cm^–1^). A medium-intensity signal is detected around the ESA maximum (ν̃_3_ = 19400 cm^–1^) for the −338 cm^–1^ beat maps, while very weak signals in the ESA region are present in the +338 cm^–1^ and ±585 cm^–1^ beat maps. These signals are due to vibrational coherences active in either S_0_ or S_1_, and their dependence on the (ν̃_1_; ν̃_3_) coordinates can be explained in terms of the displaced harmonic oscillator (DHO) model. This model predicts how a vibrational mode coupled to an electronic transition gives rise to wavepacket oscillations during *T* and thus beat map amplitude at specific excitation and detection frequency coordinates. These coordinates are predicted by double-sided Feynman diagrams (DSFDs) and marked by coded symbols superimposed on the calculated beat maps in Figure 3g–j. Due to experimental line widths in excess of a few hundred wavenumbers, signals arising from multiple DSFDs merge into broad features located between the point amplitudes predicted by individual DSFDs, rather than being detected as resolved peaks. Such an effect is especially relevant for beat maps of low-frequency vibrations such as the 338 cm^–1^ mode.

Finally, the ν̃_3_ bandwidth differences between experimental (narrow) and calculated (broad) beat maps can be rationalized on the basis of how the calculated absorptive 2DES is broadened to yield good agreement with the experimental line width. In the model, a single vibration accounts for the entirety of the experimentally observed broadening of the electronic transition. Such overestimation of the contribution of a single vibration translates to greater broadening in the calculated beat maps.

The differences in the measured beat map intensity distributions between 338 and 585 cm^–1^ modes observed here (Figure 3) are well reproduced by including a third electronic state in the DHO model of Green et al.^36^ This third state allows the inclusion of the S_1_ to S_*n*_ ESA. The 338 and 585 cm^–1^ beat maps are modeled separately, each coupled to two baths, one for electronic dephasing and one for vibrational relaxation. Each Raman-active mode is coupled to three singlet electronic states: the ground state S_0_, |*g*⟩; the first excited state S_1_, |*e*⟩; and a higher-lying excited state S_*n*_, |*f*⟩, with energies of *E*_*n*_, where *n* = {*g*, *e*, *f*}. The system Hamiltonian is thus
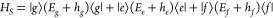
1with the nuclear contribution to the ground electronic state
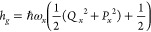
2which assumes that mode *x* = {338, 585} is harmonic with coordinate *Q*_*x*_, momentum *P*_*x*_, and frequency ω_*x*_. Coupling between the vibrational mode and electronic excited states translates to a displacement along the vibrational coordinate with respect to the ground-state minimum of Δ_*eg*_^*x*^ for S_1_ and Δ_*fg*_^*x*^ for S_*n*_ such that
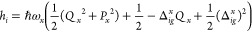
3where *i* = {*e*, *f*} and the vibrational frequency is assumed to be the same for all electronic states. The last term on the right-hand side of eq 3 corresponds to the reorganization energy for each excited-state PES. Accounting for the Stokes shift, the electronic transition frequency between S_0_ and S_1_, , is taken as the crossing point between the normalized steady-state absorption and fluorescence spectra. The electronic transition frequency between S_1_ and S_*n*_ is set so that the frequency of the ESA maximum matches the 2D spectra in Figure 1b–d, for which ν̃_3_ = 19850 cm^–1^. The displacements between the ground and first excited states of CV were determined experimentally by Batignani et al. from preresonant impulsive stimulated Raman scattering (ISRS) spectroscopy as Δ_*eg*_^585^ = 0.63 for the 585 cm^–1^ mode and Δ_*eg*_^338^ = 0.18 for the 338 cm^–1^ mode,^21^ in agreement with our experimental results, which show that the oxazine ring deformation at 585 cm^–1^ is the only Raman-active mode which is strongly resonantly enhanced, and thus significantly displaced, between S_0_ and S_1_. We thus used these literature values in our calculations. Excellent agreement between experimental and calculated 2D rephasing beat maps of CV is achieved with displacements between the ground and higher excited states of Δ_*fg*_^585^ = 0.71 for the 585 cm^–1^ mode and Δ_*fg*_^338^ = 0.26 for the 338 cm^–1^ mode. The same values yielded reasonable agreement for the nonrephasing data (shown in Figure S6) except for the −585 cm^–1^ beat map, which shows an enhancement of the SE peak which is absent from the model. As Δ_*fg*_^*x*^ = Δ_*fe*_^*x*^ + Δ_*eg*_^*x*^, this corresponds to an equal S_*n*_ ← S_1_ displacement of Δ_*fe*_^*x*^ = 0.08 for both modes. These S_1_ ← S_*n*_ displacements were determined by first calculating 2D spectra using the impulsive method described in Green et al.^43^ for a range of S_*n*_ ← S_1_ displacements of the 338 and 585 cm^–1^ modes. Rephasing and nonrephasing beat maps were obtained by applying the procedure outlined in Figure S3 to the calculated 2DES data sets. The amplitude ratio between the ESA and the GSB + SE signals of each beat map was plotted against the displacement along its normal mode coordinate to yield the data in Figures S4 and S5 and then compared to the experimental ESA/GSB + SE amplitude ratio, determined by Δ_*fe*_^*x*^, shown as a horizontal red line in Figures S4 and S5. The best match between the experimental data and impulsive calculation determined the displacements used with the computationally intense HEOM–PMA method for finite fields, obtaining the final HB2DES rephasing (Figure 3) and nonrephasing (Figure S6) beat maps. Further details on the method are given in the Supporting Information. These displacement values imply that the relative displacement in ESA, Δ_*fe*_^*x*^/Δ_*eg*_^*x*^, is much larger for the 338 cm^–1^ mode (∼0.45) than for the 585 cm^–1^ mode (∼0.13). This change is the origin of the greater relative intensity of the 338 cm^−1^ mode beat maps compared to the 585 cm^−1^ mode in the ESA region. These displacements are shown in the PESs diagram in Figure 4.

**Figure 4:**
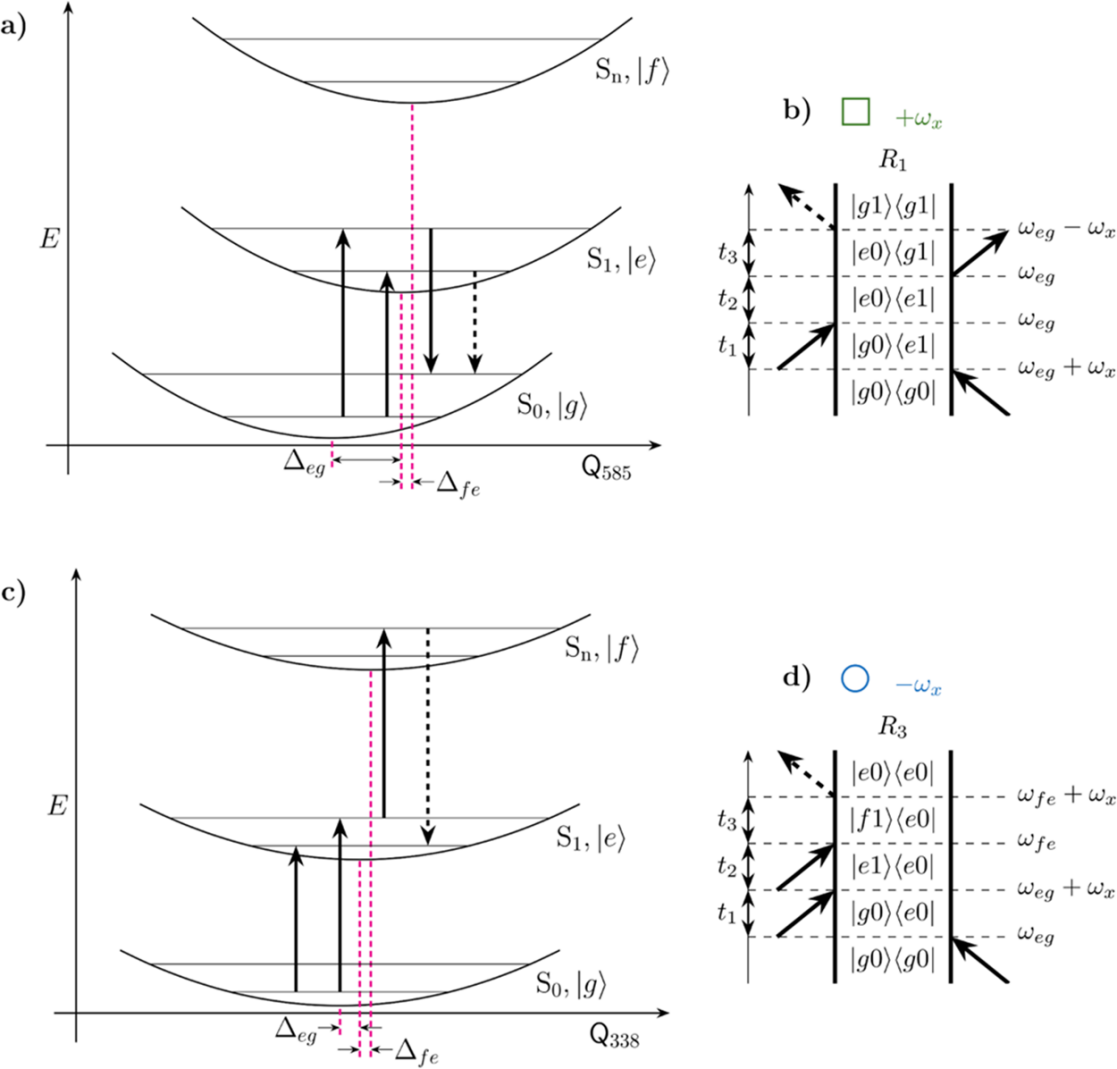
PESs along the (a) 585 and (c) 338 cm^–1^ normal mode coordinates. Dashed magenta vertical lines indicate the minima of the S_0,1,*n*_ harmonic potentials, highlighting the different displacements between S_0_,_1_,_*n*_ values along the two coordinates. Vertical solid and dashed arrows represent the four field–matter interactions yielding vibrationally coherent (a) SE and (c) ESA signals with the corresponding double-sided Feynman diagrams depicted in (b) and (d), respectively. Open squares and circles indicate vibrationally coherent SE or ESA pathways, respectively. The color of the symbols indicates the positive (green) or negative (blue) phase of the oscillation of a double-sided Feynman diagram during T.

Next we turn to the ν̃_1_ dependence of the rephasing ±338 and ±585 cm^–1^ beat maps. These are discussed in terms of the DHO model,^25,42,44^ with the excitation-frequency coordinates predicted by the coded symbols shown in Figure 3g–j, with the corresponding DSFDs shown in Figure 4b,d and in the SI (Figures S6–S9). The ν̃_1_ dependence of positive and negative beat maps (Figure 3) is due to the sign of the coherence evolving during *T.*(42) The phase-matching conditions of 2DES cause the beat map signals due to rephasing +338/+585 cm^–1^ excited-state vibrational coherences to appear blue-shifted, along the excitation axis, by at least one quantum of vibrational energy from the 0–0 electronic transition frequency at 16250 cm^–1^. This effect is illustrated by the DSFD in Figure 4b. The same phase-matching argument causes the rephasing −338/–585 cm^–1^ beat maps to be centered at the 0–0 electronic transition frequency (16250 cm^–1^), as shown by the DSFD in Figures 4d, S6, and S8. Such an effect is apparent in both the experimental (b–e) and calculated (g–j) beat maps shown in Figure 3. The opposite excitation frequency dependence holds for nonrephasing positive and negative pathways, as shown for the experimental and calculated nonrephasing ±338 and ±585 cm^–1^ beat maps reported in the Supporting Information (Figure S6).

Concerning the detection frequency dependence of the rephasing beat maps, the amplitudes of both ±338 cm^–1^ experimental (Figure 3b,c) and calculated (Figure 3g,h) beat maps are localized to the GSB + SE region (ν̃_3_ = 14000–17000 cm^–1^), with weaker features appearing in the ESA. While rephasing positive signals contain contributions from oscillatory GSB pathways, the calculated beat maps confirm that SE dominates, with the peaks centered among SE pathways above the diagonal, as a consequence of the pump spectrum located on the center of the absorption band.^36,41^ Conversely, rephasing negative beat maps with no GSB contribution is selectively reporting on S_1_ vibrational coherences. Thus, the intense feature in the ESA region of the experimental rephasing −338 cm^–1^ beat map (Figure 3c) is in very good agreement with the calculations (Figure 3h) and constitutes the unambiguous indication of a strong vibrational coherence in the S_1_ PES of CV.

The most striking discrepancy between measured and calculated beat maps is the double peak structure elongated along ν̃_1_ and evident in the GSB + SE region of the experimental rephasing ±338 cm^–1^ beat maps reported in Figure 3b,c and in the experimental nonrephasing data shown in Figure S6, which are not reproduced by the DHO model. It has been shown that interference between multiple vibrational wavepackets due to harmonic^45^ or anharmonic^46^ couplings can introduce additional complexity into 2D beat maps. The ∼500 cm^–1^ excitation frequency separation between the peaks in the experimental ±338 cm^–1^ beat maps could suggest coupling between the 338 cm^–1^ and another Raman-active mode in the S_1_ PES, which was not accounted for in the model. Another possible explanation of these features is the presence of pathways involving excitation up to three quanta of the 338 cm^–1^ mode, covered by the experimental pump spectrum (see horizontal dashed lines) but not present in the spectra calculated with a narrower Gaussian pump pulse spectrum. However, as a single-mode DHO would not reproduce the feature centered at ν̃_3_ = 15550 cm^–1^ in Figure 3c or the pattern observed below the diagonal in the nonrephasing negative data (Figure S6c), these unpredicted features are most likely due to coupling between low-frequency Raman-active vibrations.

The positive and negative experimental (Figure 3d,e) and calculated (Figure 3i,j) rephasing beat maps at 585 cm^–1^ are remarkably different from those for 338 cm^–1^. Their amplitude is mainly localized in the GSB + SE region with negligible signals detected within the ESA. Again, the selectivity of rephasing negative beat maps for S_1_ coherences allows us to assign the signals in this region of the experimental (Figure 3e) and calculated (Figure 3j) beat maps to the 585 cm^–1^ vibration modulating the energy gap of the SE transition.

These results show that the combination of HB2DES measurements and their simulation with a three-level DHO model directly reveals the displacements among the ground state, excited state, and higher excited states, through multiple resonance Raman-active vibrations. Specifically, the relative amplitude of the rephasing −338 cm^–1^ beat map in the GSB + SE and ESA regions indicates a relative displacement (Δ_*fe*_^338^/Δ_*eg*_^338^) of ∼0.45 between the S_*n*_ ← S_1_ and S_1_ ← S_0_ transitions along this normal mode coordinate. Conversely, the relative amplitude of the rephasing −585 cm^–1^ beat map is consistent with a relative displacement of ∼0.13 for S_*n*_ ← S_1_ but with a strongly displaced S_1_ ← S_0_. In other words, despite the equal S_*n*_ ← S_1_ displacement for the 338 and 585 cm^–1^ modes, the beat map amplitude in the ESA region is controlled by the magnitude of this displacement relative to the S_1_ ← S_0_ displacement, which determines the amplitude of the S_1_ wavepacket. These results are summarized in Figure 4, where the calculated displaced harmonic S_0_, S_1_, and S_*n*_ PESs along the 585 (Figure 4a) and 338 cm^–1^ (Figure 4b) normal mode coordinates are shown. Superimposed are arrows indicating the four field–dipole interactions corresponding to the rephasing positive SE and rephasing negative ESA DSFDs in Figure 4b,d, respectively. These conclusions are supported by the corresponding nonrephasing data, shown in the SI (Figure S6).

In conclusion, we reported HB2DES of CV in ethanol, where the WLC probe allows the detection of coherent dynamics in both the GSB + SE and ESA regions. Striking differences in the relative amplitudes between the (338 cm^–1^) skeletal deformation and the (585 cm^–1^) oxazine ring elongation modes in the impulsive Raman spectra of GSB + SE and ESA spectral regions were observed. A detailed analysis of the rephasing and nonrephasing positive and negative beat maps of these two Raman-active modes revealed information on the displacements among the S_0_, S_1_, and S_*n*_ potential energy surfaces. We determined small (large) displacement for 338 cm^–1^ (585 cm^–1^) between S_0_ and S_1_, in agreement with the literature. Conversely, the S_*n*_ ← S_1_ transition is equally displaced along the 338 cm^–1^ and 585 cm^–1^ coordinates, such that a greater relative excited-state displacement for the 338 cm^–1^ mode results in a larger relative wavepacket amplitude in the ESA region. Such differences in the beat map amplitude distributions between different Raman-active modes are of particular relevance when investigating the excited-state PESs of photochemically active species, in which displacements along specific vibrational coordinates may connect to the product PES.

Finally, the difference between HB2DES and other methods of monitoring displacements between excited-state vibrational modes, such as stimulated Raman scattering in the time or frequency domain, and transient 2DES spectroscopies is worth noting.^47−51^ The vibrational spectra provided by these methods could also be analyzed in terms of displacements among higher excited states. Different from these formally six-wave mixing (χ^(5)^) experiments, in which the initial actinic pulse is not (necessarily) involved in the preparation of ground- and excited-state nuclear wavepackets, coherent modulation of the S_*n*_ ← S_1_ ESA detected in the χ^(3)^ 2DES must arise from wavepacket dynamics initiated by the pump pair. Thus, in HB2DES a negligible displacement along an S_1_ ← S_0_ coordinate will suppress wavepacket dynamics in both the GSB + SE and ESA regions. Conversely, the observation of coherent dynamics in higher excited (or photochemical product) states by HB2DES directly implicates wavepacket generation initiated in the pump step. Consequently, the observation of coherences in ESA by HB2DES can indicate at least the possibility of coherent control of the photochemical processes in higher excited states.

## Experimental Methods

HB2DES measurements were carried out on a 400 mOD solution (200 μm optical path static fused silica cell, Starna Scientific Ltd.) of CV perchlorate (Exciton Inc.) in ethanol (EtOH). Dye and solvent were used as received. The 2DES spectrometer was previously described in detail.^52^ Briefly, 10% of the output of a Ti:sapphire regenerative amplifier (Spitfire Ace, Spectra-Physics) operating at 1 kHz and 800 nm seeds served as a noncollinear optical parametric amplifier (NOPA, Topas White, light conversion). The NOPA output (16400 cm^–1^, ∼800 nJ of energy per pulse pair) is compressed by a commercial folded grism compressor (Fastlite) and a pair of pump pulses with a controllable interpulse delay, and a relative carrier wave phase is generated in a commercial acousto-optical programmable dispersive filter (AOPDF, Dazzler, Fastlite). The coherence time is scanned, updating the interpulse delay and relative phase on a shot-to-shot basis, from 0 to 95 fs in 792 as steps. A three-frame phase-cycling scheme is used to obtain real and imaginary parts of the rephasing, nonrephasing, and absorptive 2D spectra.^53^ Each 2D spectrum is averaged over 180 laser shots per coherence time point. The waiting time *T* is introduced by scanning the pump pair against the probe by a retroreflector mounted on a mechanical delay stage (Physik Instrumente) in 10 fs steps from 0 to 1200 fs. The white light continuum (WLC) probe is generated by focusing a small fraction of the regenerative amplifier output in a 3 mm static sapphire plate, and it spans 13000–23000 cm^–1^. The WLC is then compressed by two pairs of dispersive mirrors (PC 1332, Ultrafast Innovations), split by a 50:50 beamsplitter and crossed at 4° with the collinear pumps at the sample position. Pump(s) and probe spot sizes are 160 and 80 μm, respectively. The signal and reference are passed through a dual-channel home-built prism-based spectrometer and recorded shot-to-shot by a pair of 1024 pixel CCD detectors (Stresing) synchronized to the amplifier and the AOPDF. The signal is referenced using an active noise reduction method proposed by Feng et al.^54^ to optimize the signal-to-noise ratio.^52^

## Supporting Information Available

Instrument response function, 2DES traces, scheme outlining the calculation of beat maps, procedure for obtaining the S_*n*_ ← S_1_ displacement from beat map amplitudes, nonrephasing beat maps, model details, and double-sided Feynman diagrams (PDF)
Transparent Peer Review report available (PDF)
