# Supplementary material for: Two-Dimensional Electronic Spectroscopy Resolves Relative Excited-State Displacements
Source: J Phys Chem Lett. 2024 Mar 6;15(10):2876–84. doi: 10.1021/acs.jpclett.3c03420 (PMC10945572; doi:10.1021/acs.jpclett.3c03420)
Supplement: Supplementary file 1 — jz3c03420_si_001.pdf [file jz3c03420_si_001.pdf]

Two-Dimensional Electronic Spectroscopy Resolves Relative Excited State Displacements

SUPPORT INFORMATION

Giovanni Bressan,[†] Dale Green,[†] Garth A. Jones,[†] Ismael A. Heisler,[‡] and Stephen R. Meech[*,†]

[†]*School of Chemistry, University of East Anglia, Norwich Research Park, Norwich, NR4 7TJ, UK*

[‡]*Instituto de Física, Universidade Federal do Rio Grande do Sul - UFRGS, Avenida Bento Gonçalves, 9500, Porto Alegre, Brazil.*

E-mail: s.meech@uea.ac.uk

Contents

Instrument Response Function **S3**

Absorptive 2DES Traces **S3**

Calculation of Beatmaps **S4**

Obtaining $S_n \leftarrow S_1$ Displacement via Beatmap Fitting **S4**

Nonrephasing Beatmaps **S7**

Model System **S9**

Hierarchical Equations of Motion **S10**

Steady State Absorption Spectrum **S13**

Equation-of-Motion Phase-Matching Approach **S14**

Vibrational Coherence Pathways **S16**

References **S21**

Instrument Response Function

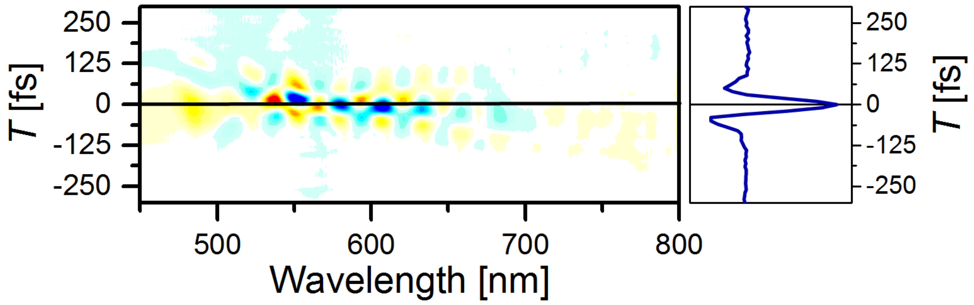

Figure S1: Instrument response function.

Absorptive 2DES Traces

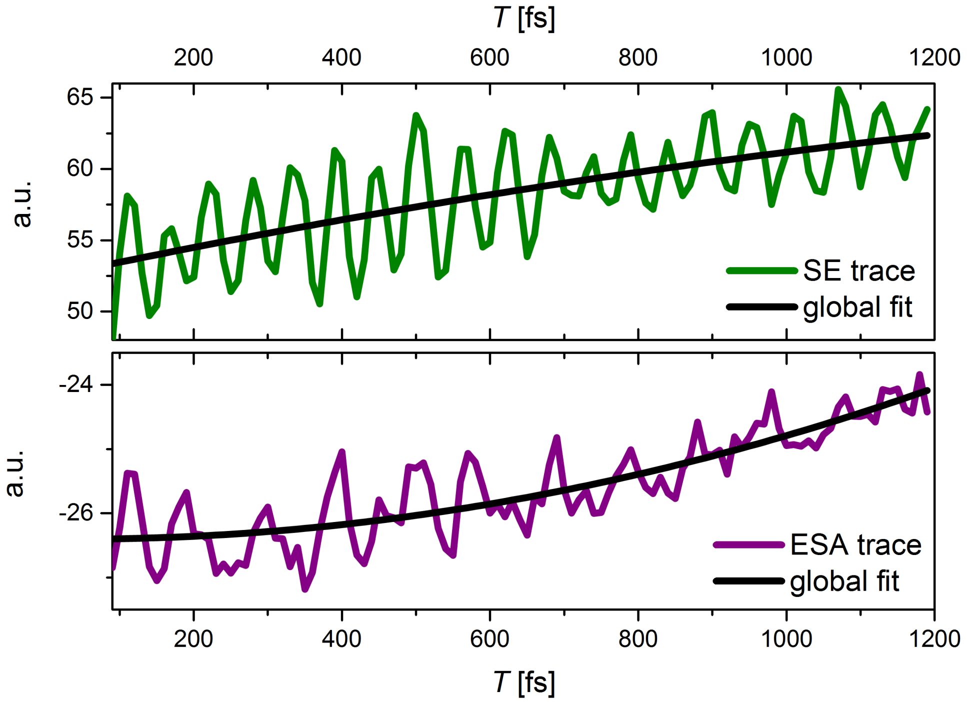

Figure S2: Absorptive 2DES traces and global fit for (top) SE and (bottom) ESA locations marked in figure 1(b) by dark green and purple squares, respectively.

Calculation of Beatmaps

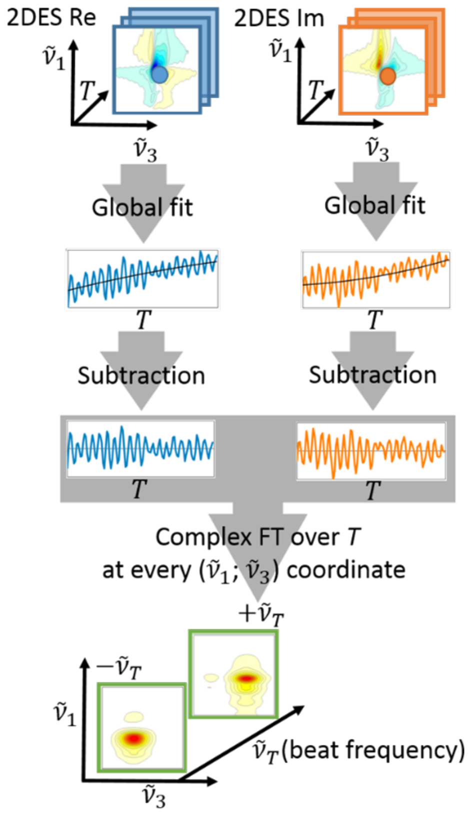

Figure S3: Scheme of the method used to recover positive and negative beatmaps from a complex-valued 2DES rephasing or nonrephasing dataset acquired in even T steps (satisfying the Nyquist sampling criterion). The Re and Im parts of the (rephasing or nonrephasing) signal are independently fit to a sum of exponential decays plus an offset to capture the "slow" population relaxation dynamics. The global fit is then subtracted from the data to isolate the residuals, corresponding to the oscillatory part of the third-order signal. The real and imaginary residuals are summed as Re + iIm to yield a complex-valued matrix which is Fourier transformed over T to obtain a 3D spectrum as a function of ν_1, ν_T and ν_3, where ν_T is the conjugate variable of the waiting time T. Beatmaps at a specific $\pm\nu_T$ are then obtained by slicing the 3D spectrum at that frequency. The beatmaps obtained by this method are shown in Figures 3 and S6.

Obtaining $S_n \leftarrow S_1$ Displacement via Beatmap Fitting

For a fixed $S_1 \leftarrow S_0$ displacement, increasing the $S_n \leftarrow S_1$ displacement increases the intensity of the ESA coherences relative to the GSB+SE coherences in the vibrational beatmaps. The

$S_n \leftarrow S_1$ displacements, Δ^x_{fe}, are therefore obtained by fitting the ratio of the maximum intensity of the ESA coherences ($\tilde{\nu}_3 \geq 18\,800\,\text{cm}^{-1}$) vs. the maximum intensity of the GSB+SE coherences ($\tilde{\nu}_3 < 18\,800\,\text{cm}^{-1}$) for the calculated beatmaps to the measured value. The values of this ratio for the rephasing $\pm 338\,\text{cm}^{-1}$ and $\pm 585\,\text{cm}^{-1}$ beatmaps in figure 3 are shown by the red lines in figure S4. The increase in this ratio for increasing Δ^x_{fe} is shown by a series of beatmaps obtained from spectra calculated using the impulsive method described in the appendix of ref. 1 which were used initially to identify an appropriate range for Δ^x_{fe}. Subsequent more intensive calculations using the equation of motion-phase matching approach for finite pulses discussed below then identified $\Delta^x_{fe} = 0.08$ as the best fit of this ratio for both the 338 and $585\,\text{cm}^{-1}$ modes. Figure S5 shows the equivalent for the nonrephasing beatmaps which also supports the fit of $\Delta^x_{fe} = 0.08$ for both the 338 and $585\,\text{cm}^{-1}$ modes.

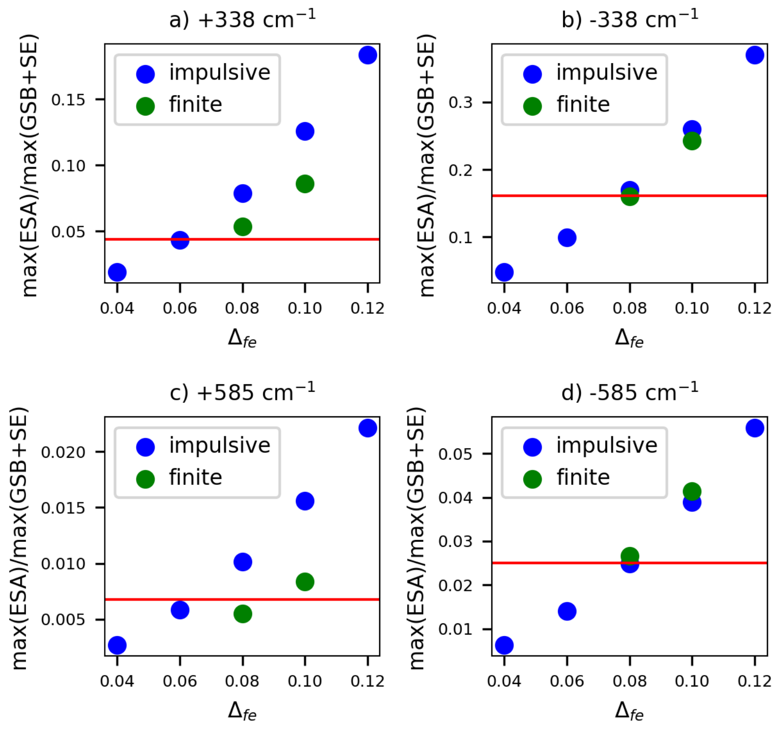

Figure S4: Rephasing a) $+338\,\text{cm}^{-1}$ b) $-338\,\text{cm}^{-1}$ c) $+585\,\text{cm}^{-1}$ d) $-585\,\text{cm}^{-1}$ beatmap maximum intensity ratio of the ESA region ($\tilde{\nu}_3 \geq 18\,800\,\text{cm}^{-1}$) vs. GSB+SE region for 2D spectra calculated impulsively (blue) and with finite pulses (green) compared to the experimental ratio (red line) to fit Δ_{fe}.

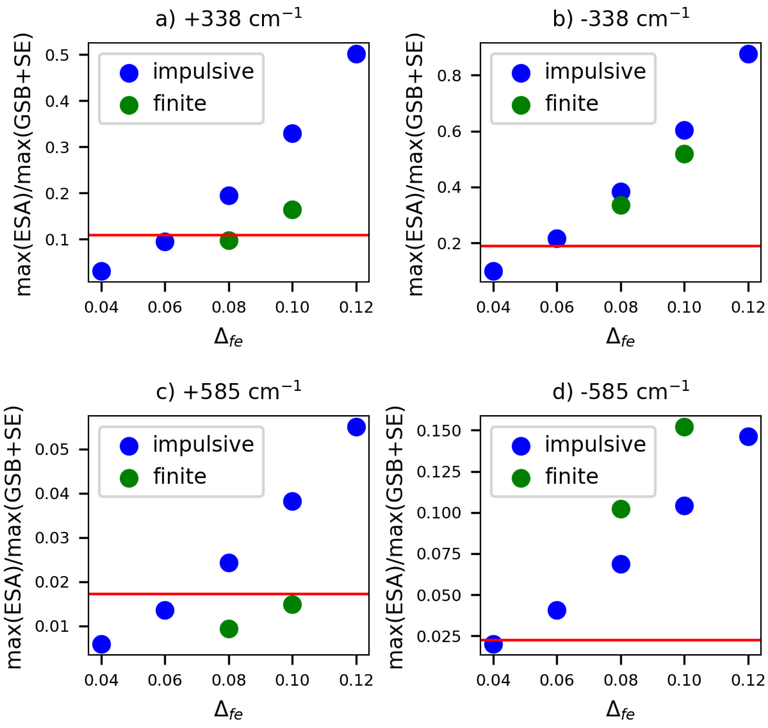

Figure S5: Nonrephasing +338 cm^{-1} b) −338 cm^{-1} c) +585 cm^{-1} d) −585 cm^{-1} beatmap maximum intensity ratio of the ESA region ($\tilde{\nu}_3 \geq 18\,800$ cm^{-1}) vs. GSB+SE region for 2D spectra calculated impulsively (blue) and with finite pulses (green) compared to the experimental ratio (red line) to fit Δ_{fe}.

Nonrephasing Beatmaps

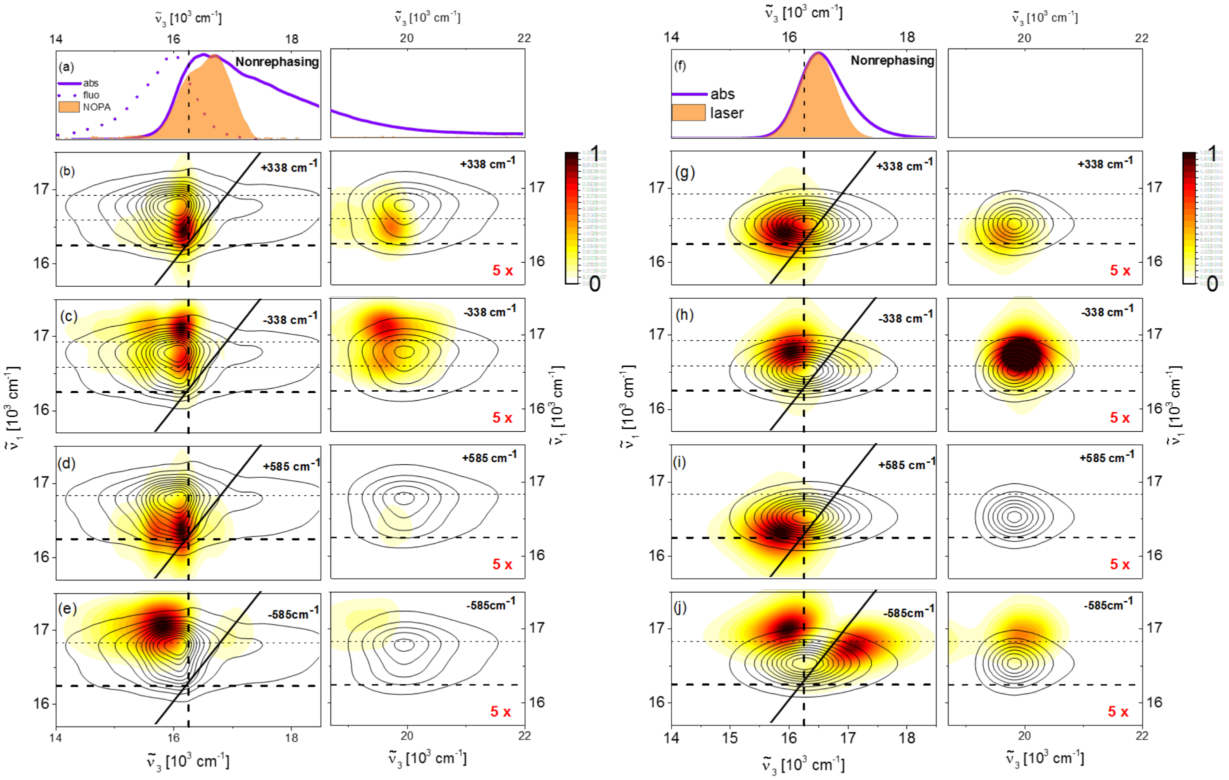

Figure S6: (a) Normalised steady-state absorption (solid) and emission (dashed) spectra of CV along with the NOPA pump spectrum. The probe axes are broken at 18 400 cm^{-1} to highlight that the beatmap amplitudes are 5x rescaled in the ESA regions. (b-c) Nonrephasing positive and negative beatmaps of the 338 cm^{-1} Raman active mode are shown as white-yellow-red heatmaps and are all normalised to 1. (d-e) Same as b-c for the 585 cm^{-1} Raman active mode. Contour lines showing the real part of the experimental absorptive 2D spectrum (T = 500 fs) are overlaid in b-e. Vertical and horizontal thick dashed lines are drawn at the 0-0 electronic transition frequency (16 250 cm^{-1}) and horizontal thin dashed lines are drawn at $+1, 2$ quanta of vibrational excitation. (f) are the pump and CV steady-state absorption spectra used in the simulated 2DES of CV. (g, h) are the same as (b, c) and (i, j) are the same as (d, e) for the simulated nonrephasing positive and negative beatmaps of the 338 and 585 cm^{-1} modes. Contour lines showing the real part of the calculated absorptive 2D spectrum (T = 500 fs) are overlaid in g-j.

The nonrephasing beatmaps in fig. S6 show the same features as the rephasing beatmaps in figure 3. The experimental beatmaps all have peaks in the GSB+SE region which are significantly more intense than peaks due to ESA coherences. However, the much greater intensity of ESA coherences in the ±338 cm^{-1} beatmaps compared to the ±585 cm^{-1} beatmaps again

indicates a much greater relative displacement for the $338\,\mathrm{cm}^{-1}$ mode than for the $585\,\mathrm{cm}^{-1}$ mode. The calculated beatmaps show good agreement with the experimental data, with peaks aligned with respect to the excitation axis, $\tilde{\nu}_1$, although limited in this axis by the slightly narrower Gaussian pump spectrum. The calculated beatmaps reproduce the much greater ESA intensity for the $338\,\mathrm{cm}^{-1}$ mode, but the ESA peak in the calculated $-585\,\mathrm{cm}^{-1}$ beatmap in fig. S6(j) is more intense than in the experimental beatmap. This is a consequence of the enhancement of the SE peak at $\tilde{\nu}_3 = 16\,000\,\mathrm{cm}^{-1}$ in fig. S6(e) compared with the peak at $\tilde{\nu}_3 = 17\,000\,\mathrm{cm}^{-1}$ which is absent from the model, where these peaks have the same intensity in fig. S6(j). Similarly, the multiple peaks in the GSB+SE region of the $\pm 338\,\mathrm{cm}^{-1}$ experimental beatmaps, as in the rephasing beatmaps, are not observed in the calculated beatmaps as the model accounts for a single vibrational mode only. Thus the model cannot account for coupling with other vibrational modes and overestimates the broadening of this mode in an effort to match the experimental lineshape which is in fact the result of multiple vibronic progressions.

Model System

The nuclear Hamiltonians and potential energy surfaces described in the main text are presented using a dimensionless coordinate system of,

$$P_x = \left(\sqrt{\hbar\omega_x m_x}\right)^{-1} p_x, \tag{S1}$$

$$Q_x = \left(\sqrt{\frac{\omega_x m_x}{\hbar}}\right) q_x, \tag{S2}$$

where m_x, ω_x, p_x, and q_x are respectively the mass, frequency, momentum and coordinate of the vibrational mode identified by $x = \{338, 585\}$. The corresponding vibrational creation and annihilation operators are, respectively,

$$b_x^\dagger = \frac{1}{\sqrt{2}}\left(Q_x - iP_x\right), \tag{S3}$$

$$b_x = \frac{1}{\sqrt{2}}\left(Q_x + iP_x\right), \tag{S4}$$

such that,

$$Q_x = \frac{b_x + b_x^\dagger}{\sqrt{2}}, \tag{S5}$$

$$P_x = \frac{b_x - b_x^\dagger}{i\sqrt{2}}. \tag{S6}$$

In second quantized form, the nuclear Hamiltonians for the three electronic states are therefore,

$$h_g = E_g + \hbar\omega_x(b_x^\dagger b_x + 1/2), \tag{S7}$$

$$h_e = E_e + \hbar\omega_x(b_x^\dagger b_x + \frac{1}{2} - \frac{\Delta_{eg}^x}{\sqrt{2}}(b_x^\dagger + b_x) + \frac{1}{2}(\Delta_{eg}^x)^2), \tag{S8}$$

$$h_f = E_f + \hbar\omega_x(b_x^\dagger b_x + \frac{1}{2} - \frac{\Delta_{fg}^x}{\sqrt{2}}(b_x^\dagger + b_x) + \frac{1}{2}(\Delta_{fg}^x)^2). \tag{S9}$$

For both the $338\,\mathrm{cm}^{-1}$ and $585\,\mathrm{cm}^{-1}$ models, the system Hamiltonian is constructed using equations S7 - S9 with 10 vibrational levels per electronic state prior to diagonalization into

the adiabatic basis and truncation to 5 vibrational levels to reduce computation time.

Electronic transitions from $S_1 \leftarrow S_0$ and $S_n \leftarrow S_1$ are dipole-allowed such that the electronic dipole moment operator,

$$\hat{\mu}_{\text{el}} = \vec{\mu}_{eg}(|e\rangle\langle g| + |g\rangle\langle e|) + \vec{\mu}_{fe}(|f\rangle\langle e| + |e\rangle\langle f|), \tag{S10}$$

where the two transition dipole moments are assumed to be collinear and of equal magnitude, $\vec{\mu}_{eg} = \vec{\mu}_{fe}$. The total dipole moment operator $\hat{\mu} = \hat{\mu}_{\text{el}} \otimes I_{\text{vib}}$ where $I_{\text{vib}} = \sum_\nu |\nu\rangle\langle\nu|$ is the identity operator over the vibrational levels. All system operators are transformed into the adiabatic basis and truncated to match the Hamiltonian.

Hierarchical Equations of Motion

The environment is modelled as an ensemble of harmonic oscillators separated into two baths with the total spectral density,

$$J(\omega) = J_{\text{el}}(\omega) + J_{\text{vib}}(\omega). \tag{S11}$$

The first bath represents the electronic interaction with the solvent environment, introducing fluctuations in the electronic transition energies responsible for electronic dephasing. The second represents the vibrational environment, by which we refer to all intermolecular and other intramolecular modes, enabling vibrational relaxation and dephasing. Both the electronic and vibrational baths are assumed to have overdamped spectral densities, approximated with the Lorentz-Drude form,

$$J_n(\omega) = 2\eta_n \frac{\omega\Lambda_n}{\omega^2 + \Lambda_n^2}, \tag{S12}$$

where η_n is the reorganisation energy of the bath $n = \{\mathrm{el}, \mathrm{vib}\}$ and Λ_n^{-1} is the correlation time. The inverse temperature, $\beta = (k_\mathrm{B}T)^{-1}$, is assumed the same for both baths with $T = 298\,\mathrm{K}$. The electronic bath, with $\eta_\mathrm{el}/2\pi c = 300\,\mathrm{cm}^{-1}$ and $\Lambda_\mathrm{el}/2\pi c = 100\,\mathrm{cm}^{-1}$, introduces significant inhomogeneous broadening that engulfs the vibronic progression and induces rapid spectral diffusion.[1] The weaker, more homogeneous vibrational bath, with $\eta_\mathrm{vib}/2\pi c = 40\,\mathrm{cm}^{-1}$ and $\Lambda_\mathrm{vib}/2\pi c = 200\,\mathrm{cm}^{-1}$, results in the slow decay of vibrational wavepackets such that beatings survive well beyond $T = 1\,\mathrm{ps}$, as seen in the experimental HB2DES spectra (Figure 2).

A hierarchy of equations of motion is derived for the auxiliary density operators (ADOs), $\rho_\mathbf{j}$, as,[1,2]

$$\begin{aligned} \dot{\rho}_\mathbf{j}(t) \quad = \quad & -\left(\frac{i}{\hbar}H_\mathrm{S}'^{\times} + \sum_{n=1}^{N}\sum_{k=0}^{M} j_{nk}\nu_{nk}\right)\rho_\mathbf{j}(t) - i\sum_{n=1}^{N}\sum_{k=0}^{M} B_n^{\times}\rho_{j_{nk}^{+}}(t) \\ & -i\sum_{n=1}^{N}\sum_{k=0}^{M} j_{nk}\left(c_{nk}B_n\rho_{j_{nk}^{-}}(t) - c_{nk}^{*}\rho_{j_{nk}^{-}}(t)B_n\right) \\ & -\sum_{n=1}^{N}\left(\frac{2\eta_n}{\hbar\beta\Lambda_n} - \eta_n\cot\left(\frac{\hbar\beta\Lambda_n}{2}\right) - \sum_{k=1}^{M}\frac{c_{nk}}{\nu_{nk}}\right)B_n^{\times}B_n^{\times}\rho_\mathbf{j}(t), \end{aligned} \tag{S13}$$

where $H_\mathrm{S}'^{\times}\rho = [H_\mathrm{S}', \rho]$ denotes the commutator of the density matrix and the renormalised system Hamiltonian,

$$H_\mathrm{S}' = H_\mathrm{S} + \sum_{n}^{N}\eta_n B_n^2 \tag{S14}$$

which counters the energy shift induced by the system-bath coupling.[3,4]

The reduced density operator of the system corresponds to $\rho_\mathbf{0}$, where all elements of $\mathbf{j}$ are equal to zero. Each ADO is uniquely identified by the $2(M+1)$-dimensional vector $\mathbf{j}$, with elements, j_{nk}, where $n = \{\mathrm{el}, \mathrm{vib}\}$, $k = 0$ corresponds to the primary Brownian oscillator mode and $k > 0$ are Matsubara axes. The frequencies, $\nu_{nk}; k = 0, 1, 2, ..., M$, and

the coefficients, c_{nk}, are given by,

$$\nu_{n0} = \Lambda_n, \tag{S15}$$

$$\nu_{nk} = \frac{2\pi k}{\hbar\beta}, \tag{S16}$$

$$c_{n0} = \eta_n\Lambda_n\left(\cot\left(\frac{\hbar\beta\Lambda_n}{2}\right) - i\right), \tag{S17}$$

$$c_{nk} = \frac{4\eta_n\Lambda_n}{\hbar\beta}\left(\frac{\nu_{nk}}{\nu_{nk}^2 - \Lambda_n^2}\right). \tag{S18}$$

The hierarchy is terminated by enforcing the Markovian limit as ξ, which determines the number of Matsubara axes for each bath, M, and the maximum value of j_{nk} for each axis as,[1,2]

$$\frac{2(M+1)\pi}{\hbar\beta} > \xi, \tag{S19}$$

$$\sum_n \sum_{k=0}^{M} j_{nk}\nu_{nk} > \xi. \tag{S20}$$

This termination criterion is suitable as long as ξ is significantly larger than the dissipation rate of the bath, Λ_n. Any evolution involving frequencies greater than the Markovian limit is too rapid to be affected by non-Markovian feedback and the equation of motion for terminating ADOs is approximated to the Liouville-von Neumann form, ignoring any further influence from the bath.

$$\dot{\rho}_{\mathbf{j}}(t) \simeq -\frac{i}{\hbar}H_{\mathrm{S}}^{\prime\times}\rho_{\mathbf{j}}(t). \tag{S21}$$

For the above bath parameters, convergence is achieved with a Markovian limit of $\xi = 2000\,\mathrm{cm}^{-1}$, which results in a hierarchy with 729 ADOs.

The diagonal coupling operator for the electronic bath,

$$B_{\mathrm{el}} = (|e\rangle\langle e| + 2\,|f\rangle\langle f|) \otimes \sum_\nu |\nu\rangle\langle\nu|, \tag{S22}$$

where $\sum_\nu |\nu\rangle\langle\nu|$ is the identity operator over the vibrational levels, causes the electronic

transition frequencies to fluctuate. The factor of 2 ensures the rate of dephasing between $\mathrm{S_0}$ and $\mathrm{S_1}$ is the same as that between $\mathrm{S_1}$ and $\mathrm{S_n}$.

The off-diagonal coupling operator for the vibrational bath,

$$B_{\mathrm{vib}} = (|g\rangle\langle g| + |e\rangle\langle e| + |f\rangle\langle f|) \otimes \frac{(b_x + b_x^\dagger)}{\sqrt{2}}, \tag{S23}$$

causes vibrational relaxation and dephasing for all electronic states, but results in a blueshift of the vibrational frequency of the system which is corrected by adjusting the mode frequency in the system Hamiltonian,[5]

$$\omega_x = \omega_x' - \eta_{\mathrm{vib}}, \tag{S24}$$

where $\omega_x'/2\pi c = 338$ or $585\,\mathrm{cm}^{-1}$.

Spectra are calculated from correlated initial conditions obtained after a 2 ps equilibration period,[6,7] prior to which the reduced density matrix of the system, $\rho_{\mathbf{0}}$, is initially defined as a Boltzmann distribution over the vibrational levels of the ground electronic state.

Steady State Absorption Spectrum

The steady state absorption spectrum is calculated in the impulsive limit as the Fourier transform of the first order molecular response function,[8,9]

$$\sigma_A(\omega) = \int_0^\infty \mathrm{d}t e^{i\omega t} \mathbf{Tr}_g \left(\hat{\mu} \hat{G}(t, t_0)[\hat{\mu}, \rho_{\mathbf{j}}(-\infty)] \right), \tag{S25}$$

where the trace is taken over the ground electronic state of $\rho_{\mathbf{0}}$ only and $\hat{G}(t, t_0)$ corresponds to propagation using the HEOM, after application of the commutator to all equilibrated ADOs.

Equation-of-Motion Phase-Matching Approach

2D electronic spectroscopy involves three system-field interactions, with the first and second separated by the coherence time, τ, and the second and third by the population time, T, producing a third order polarization which dephases over the final time period, $t > 0$; fig. S7.[10]

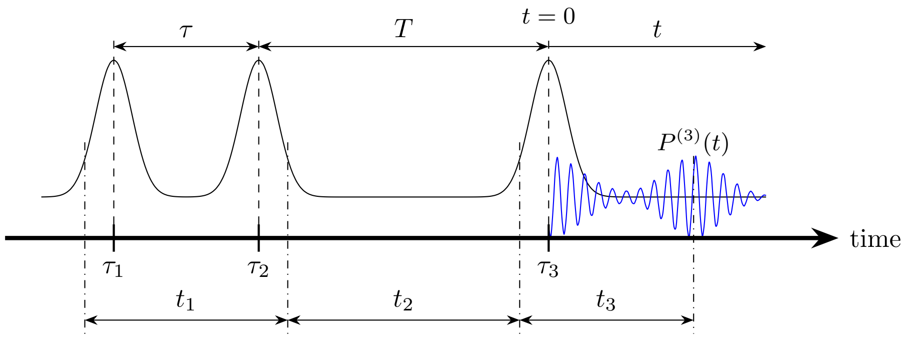

Figure S7: Pulse sequence for 2D electronic spectroscopy.

The system-field interaction Hamiltonian in the dipole approximation is split into three portions,[11,12]

$$H_{\mathrm{SF}}(t) = -\hat{\mu}\mathcal{E}(\mathbf{r}, t) = -\sum_{m=1}^{3} V_m(t) + c.c., \tag{S26}$$

where $m = 1, 2$ corresponds to the narrow pump pulse, $m = 3$ the white-light probe pulse and *c.c.* is the complex conjugate. Each pulse portion,

$$V_m(t) = \left(\hat{\mu}\chi_m E'_m(t - \tau_m) \exp(i\mathbf{k}_m \cdot \mathbf{r} - i\omega_m t)\right), \tag{S27}$$

with frequency $\omega_m = 2\pi\nu_m$, wavevector $\mathbf{k}_m$, electric field strength, χ_m, and field envelope, $E'_m(t - \tau_m)$, centred at τ_m, is assumed to be Gaussian with FWHM τ_p,[13,14]

$$E'_m(t - \tau_m) = \exp\left(\frac{-4\ln 2(t - \tau_m)^2}{\tau_p^2}\right). \tag{S28}$$

The pump spectrum is modelled using a Gaussian pulse with FWHM of $\tau_p = 40\,\mathrm{fs}$ centred at $\omega_{1,2} = 16\,500\,\mathrm{cm}^{-1}$ with field strength $\chi_{1,2} = 1 \times 10^7\,\mathrm{V\,m}^{-1}$, and the weaker white-light

probe by a Gaussian with FWHM of $\tau_p = 2\,\mathrm{fs}$ centred at $\omega_3 = 16\,000\,\mathrm{cm}^{-1}$ with field strength of $\chi_3 = 1 \times 10^5\,\mathrm{V\,m}^{-1}$. The polarisation vectors of the pulses are assumed parallel to the electronic transition dipole moments of the system.

The equation-of-motion phase-matching approach (EOM-PMA) involves propagation of seven auxiliaries which correspond to variations in the number and order of pulse interactions.

$$\mathcal{L}_1\rho_1(t) = -\frac{i}{\hbar}\left[H_\mathrm{S} - V_1(t) - V_2^\dagger(t) - V_3^\dagger(t), \rho_1(t)\right], \tag{S29}$$

$$\mathcal{L}_2\rho_2(t) = -\frac{i}{\hbar}\left[H_\mathrm{S} - V_1(t) - V_2^\dagger(t), \rho_2(t)\right], \tag{S30}$$

$$\mathcal{L}_3\rho_3(t) = -\frac{i}{\hbar}\left[H_\mathrm{S} - V_1(t) - V_3^\dagger(t), \rho_3(t)\right], \tag{S31}$$

$$\mathcal{L}_4\rho_4(t) = -\frac{i}{\hbar}\left[H_\mathrm{S} - V_1(t), \rho_4(t)\right], \tag{S32}$$

$$\mathcal{L}_5\rho_5(t) = -\frac{i}{\hbar}\left[H_\mathrm{S} - V_2^\dagger(t) - V_3^\dagger(t), \rho_5(t)\right], \tag{S33}$$

$$\mathcal{L}_6\rho_6(t) = -\frac{i}{\hbar}\left[H_\mathrm{S} - V_2^\dagger(t), \rho_6(t)\right], \tag{S34}$$

$$\mathcal{L}_7\rho_7(t) = -\frac{i}{\hbar}\left[H_\mathrm{S} - V_3^\dagger(t), \rho_7(t)\right]. \tag{S35}$$

Combination of these auxiliaries then isolates the third order polarization produced by a single interaction with each pulse in the phase-matched direction, $\mathbf{k}_s$,[11]

$$P^{(3)}_{\mathbf{k}_s}(\tau, T, t) = \mathbf{Tr}\big(\hat{\mu}\,(\rho_1(t) - \rho_2(t) - \rho_3(t) + \rho_4(t) - \rho_5(t) + \rho_6(t) + \rho_7(t))\big) + c.c. \tag{S36}$$

where the complex conjugate term accounts for the complementary set of Liouville pathways. The number of auxiliaries required can be reduced by enforcing the rotating wave approximation,[12,15] but here the full seven are used.

Calculation of the rephasing, $\mathbf{k}_\mathrm{R} = -\mathbf{k}_1 + \mathbf{k}_2 + \mathbf{k}_3$, or nonrephasing, $\mathbf{k}_\mathrm{NR} = \mathbf{k}_1 - \mathbf{k}_2 + \mathbf{k}_3$, polarization is determined by the time ordering of the pump pulses, where positive coherence times, $\tau > 0$, correspond to the rephasing pathways, whilst negative coherence times, $\tau < 0$, exchange the order of the first two field interactions producing the nonrephasing pathways.

It is critical to correctly account for the phase difference between the pulses, where

calculation of the spatial phase can be simplified in the knowledge that,

$$\mathbf{k_1} \cdot \mathbf{r} = \omega(\tau_1), \tag{S37}$$

$$\mathbf{k_2} \cdot \mathbf{r} = \omega(\tau_1 + \tau), \tag{S38}$$

$$\mathbf{k_3} \cdot \mathbf{r} = \omega(\tau_1 + \tau + T), \tag{S39}$$

where τ_1 is the time from the beginning of the simulation to the centre of the first pulse (fig. S7).

2D electronic spectra are then obtained by Fourier transform with respect to both τ and t. The rephasing spectra, S_{R}, require the inverse transform with respect to the coherence time ($\propto \exp(-i\omega_\tau \tau)$), whilst the nonrephasing, S_{NR}, require the forwards transform ($\propto \exp(+i\omega_\tau \tau)$), reflecting the reversal in time ordering,[14,16]

$$S_{\mathrm{R}}(\omega_\tau, T, \omega_t) = \int_0^\infty \mathrm{d}t \int_0^\infty \mathrm{d}\tau e^{-i\omega_\tau \tau} e^{i\omega_t t} i P^{(3)}_{\mathbf{k}_{\mathrm{R}}}(\tau, T, t), \tag{S40}$$

$$S_{\mathrm{NR}}(\omega_\tau, T, \omega_t) = \int_0^\infty \mathrm{d}t \int_0^\infty \mathrm{d}\tau e^{i\omega_\tau \tau} e^{i\omega_t t} i P^{(3)}_{\mathbf{k}_{\mathrm{NR}}}(\tau, T, t). \tag{S41}$$

Vibrational Coherence Pathways

2D spectra were calculated in steps of 20 fs up to $T = 900$ fs. Non-oscillatory population pathways are removed via global fit along the population time, T. Fourier transform of the residuals then produces vibrational beating maps which distinguish positively and negatively oscillating coherence pathways as in figure S3. We identify positive coherences, $\propto e^{+i\omega_x T}$, in green and negative coherences, $\propto e^{-i\omega_x T}$, in blue such that,

$$|g0\rangle\langle g1| \propto e^{+i\omega_x T}; \qquad |g1\rangle\langle g0| \propto e^{-i\omega_x T}$$

The coherence pathways for ground state bleach (GSB), stimulated emission (SE) and

excited state absorption (ESA) processes are labelled using colour-coded symbols, as defined in table S1.

Table S1: Symbol Key for Liouville pathways.

	GSB	SE	ESA
Positive Coherence ($+\omega_x$)	△	□	○
Negative Coherence ($-\omega_x$)	△	□	○

Global fit of the calculated 2D electronic spectra required two exponential decay components for both the 338 cm^{-1} and 585 cm^{-1} models, one fast (ca. 50 fs) and one long (> 1 ns), as well as trimming of the first $T = 100$ fs to remove distortion at early times due to the coherent artefact.

The vibrational coherence pathways labelled in figure 3 are listed in figures S8–S11.

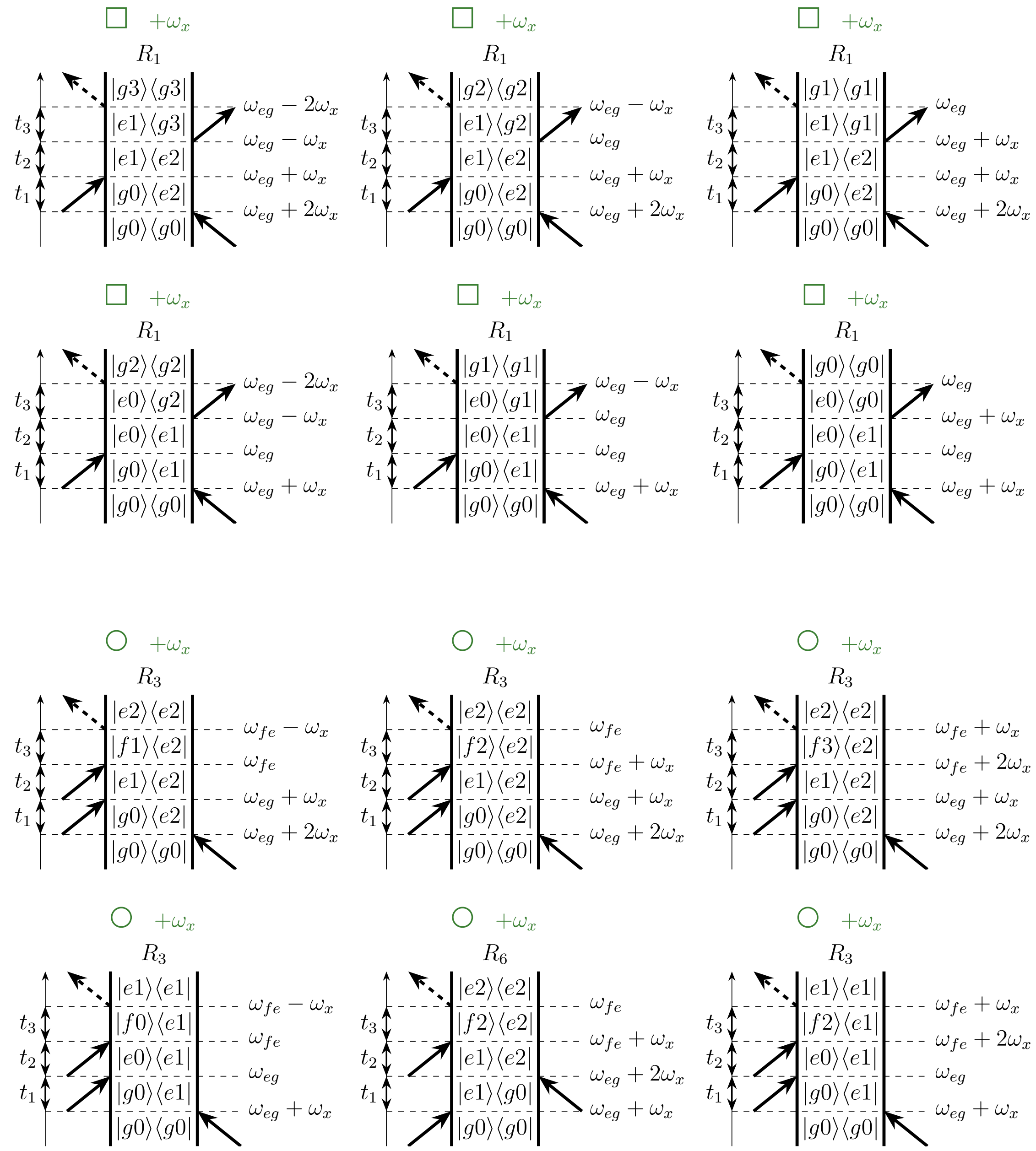

Figure S8: Rephasing $\omega_T/2\pi c = +338\,\mathrm{cm}^{-1}$ Liouville pathways.

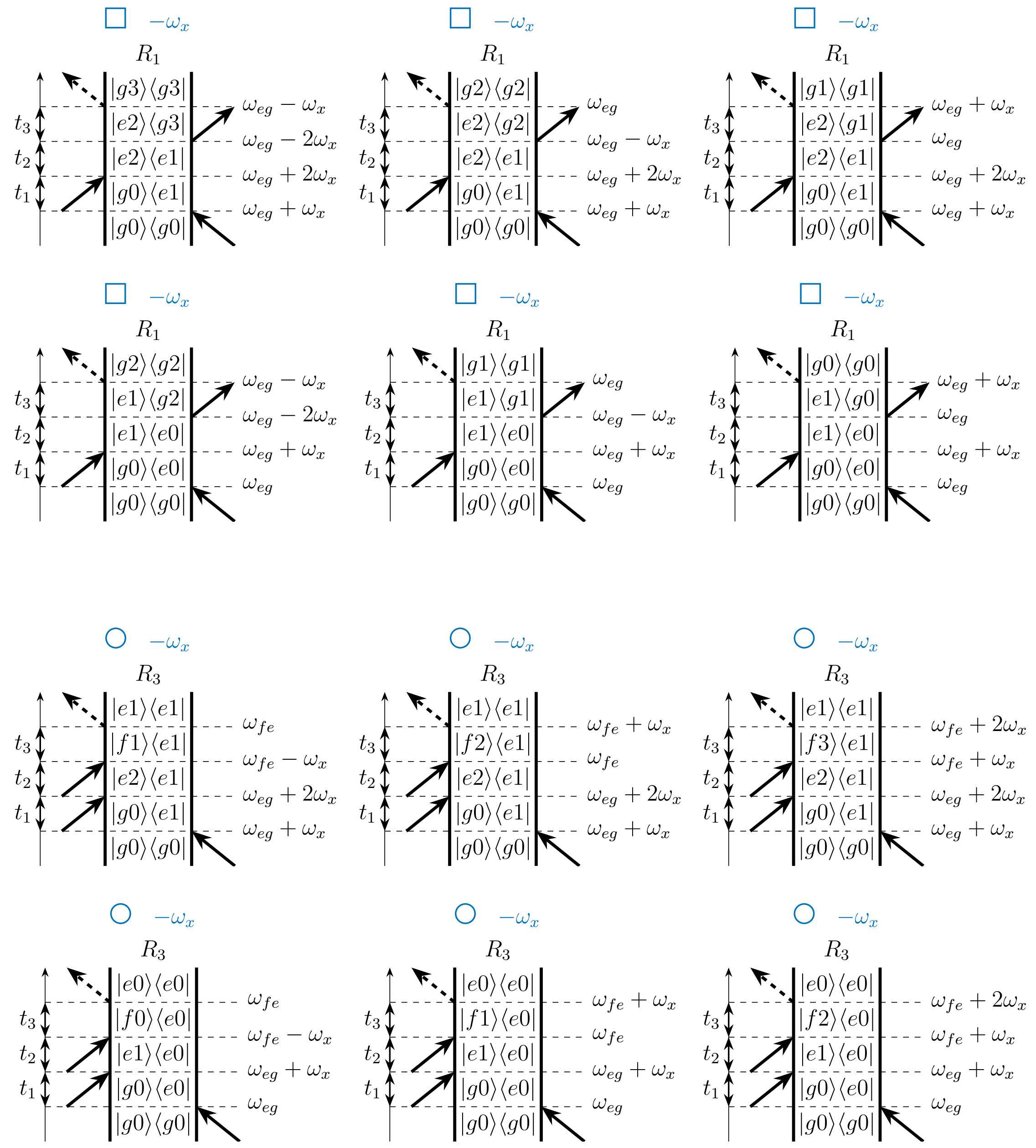

Figure S9: Rephasing $\omega_T/2\pi c = -338\,\mathrm{cm}^{-1}$ Liouville pathways.

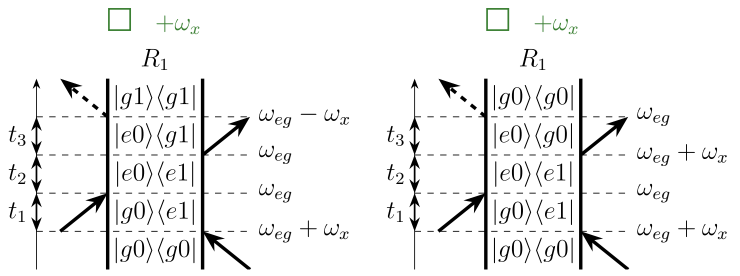

Figure S10: Rephasing $\omega_T/2\pi c = +585\,\mathrm{cm}^{-1}$ Liouville pathways.

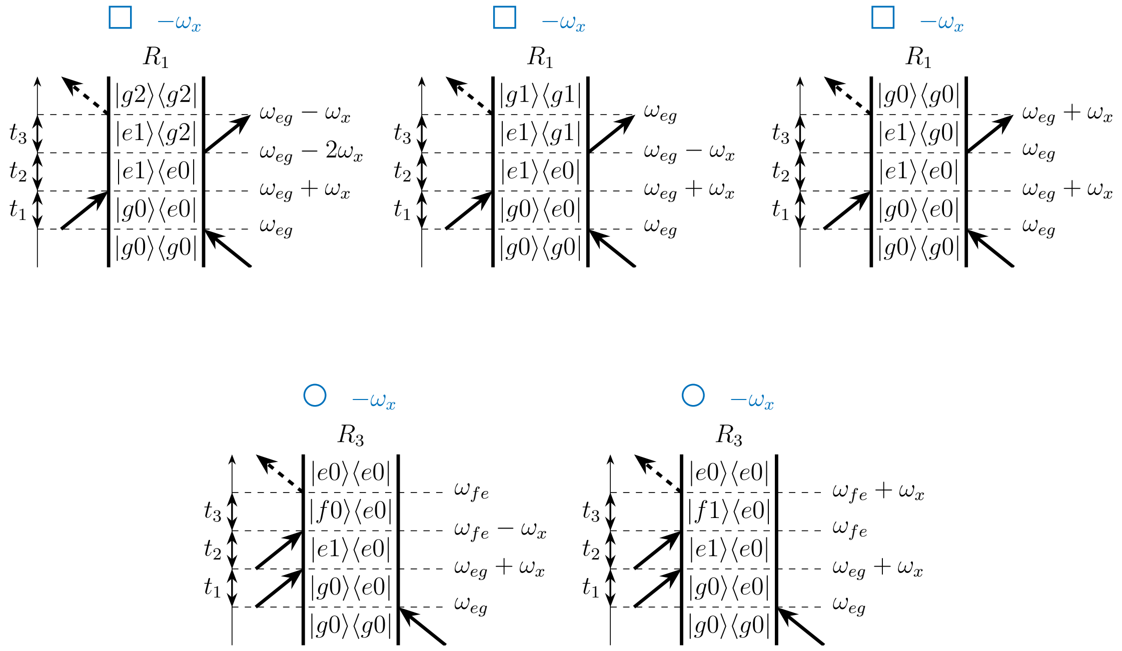

Figure S11: Rephasing $\omega_T/2\pi c = -585\,\mathrm{cm}^{-1}$ Liouville pathways.

References

(1) Green, D.; Humphries, B. S.; Dijkstra, A. G.; Jones, G. A. Quantifying Non-Markovianity in Underdamped versus Overdamped Environments and its Effect on Spectral Lineshape. *J. Chem. Phys.* **2019**, *151*, 174112.

(2) Dijkstra, A. G.; Prokhorenko, V. I. Simulation of Photo-Excited Adenine in Water with a Hierarchy of Equations of Motion Approach. *J. Chem. Phys.* **2017**, *147*, 064102.

(3) Dijkstra, A. G.; Tanimura, Y. Linear and Third- and Fifth-Order Nonlinear Spectroscopies of a Charge Transfer System Coupled to an Underdamped Vibration. *J. Chem. Phys.* **2015**, *142*, 212423.

(4) Tanimura, Y. Numerically "Exact" Approach to Open Quantum Dynamics: The Hierarchical Equations of Motion (HEOM). *J. Chem. Phys.* **2020**, *153*, 020901.

(5) Bennett, D. I. G.; Malý, P.; Kreisbeck, C.; van Grondelle, R.; Aspuru-Guzik, A. Mechanistic Regimes of Vibronic Transport in a Heterodimer and the Design Principle of Incoherent Vibronic Transport in Phycobiliproteins. *J. Phys. Chem. Lett.* **2018**, *9*, 2665–2670.

(6) Ishizaki, A.; Tanimura, Y. Dynamics of a Multimode System Coupled to Multiple Heat Baths Probed by Two-Dimensional Infrared Spectroscopy. *J. Phys. Chem. A* **2007**, *111*, 9269–9276.

(7) Tanimura, Y. Reduced Hierarchical Equations of Motion in Real and Imaginary Time: Correlated Initial States and Thermodynamic Quantities. *J. Chem. Phys.* **2014**, *141*, 044114.

(8) Chen, L.; Zheng, R.; Shi, Q.; Yan, Y. Optical Line Shapes of Molecular Aggregates: Hierarchical Equations of Motion Method. *J. Chem. Phys.* **2009**, *131*, 094502.

(9) Tanimura, Y. Reduced Hierarchy Equations of Motion Approach with Drude plus Brownian Spectral Distribution: Probing Electron Transfer Processes by means of Two-Dimensional Correlation Spectroscopy. *J. Chem. Phys.* **2012**, *137*, 22A550.

(10) Biswas, S.; Kim, J.; Zhang, X.; Scholes, G. D. Coherent Two-Dimensional and Broadband Electronic Spectroscopies. *Chem. Rev.* **2022**, *122*, 4257–4321.

(11) Gelin, M. F.; Egorova, D.; Domcke, W. Efficient Method for the Calculation of Time- and Frequency-Resolved Four-Wave Mixing Signals and its Application to Photon-Echo Spectroscopy. *J. Chem. Phys.* **2005**, *123*, 164112.

(12) Gelin, M. F.; Egorova, D.; Domcke, W. Efficient Calculation of Time- and Frequency-Resolved Four-Wave-Mixing Signals. *Acc. Chem. Res.* **2009**, *42*, 1290–1298.

(13) Sharp, L. Z.; Egorova, D.; Domcke, W. Efficient and Accurate Simulations of Two-Dimensional Electronic Photon-Echo Signals: Illustration for a Simple Model of the Fenna-Matthews-Olson Complex. *J. Chem. Phys.* **2010**, *132*, 014501.

(14) Leng, X.; Yue, S.; Weng, Y.-X.; Song, K.; Shi, Q. Effects of Finite Laser Pulse Width on Two-Dimensional Electronic Spectroscopy. *Chem. Phys. Lett.* **2017**, *667*, 79–86.

(15) Cheng, Y.-C.; Lee, H.; Fleming, G. R. Efficient Simulation of Three-Pulse Photon-Echo Signals with Application to the Determination of Electronic Coupling in a Bacterial Photosynthetic Reaction Center. *J. Phys. Chem. A* **2007**, *111*, 9499–9508.

(16) Cheng, Y.-C.; Engel, G. S.; Fleming, G. R. Elucidation of Population and Coherence Dynamics using Cross-Peaks in Two-Dimensional Electronic Spectroscopy. *Chem. Phys.* **2007**, *341*, 285–295.
