# Supplementary material for: Two-Dimensional Electronic Spectroscopy Resolves Relative Excited-State Displacements
Source: J Phys Chem Lett. 2024 Mar 6;15(10):2876–84. doi: 10.1021/acs.jpclett.3c03420 (PMC10945572; doi:10.1021/acs.jpclett.3c03420)
Supplement: Supplementary file 2 — jz3c03420_si_002.pdf [file jz3c03420_si_002.pdf]

jz-2023-03420m.R1

Name: Peer Review Information for "Two-Dimensional Electronic Spectroscopy Resolves Relative Excited State Displacements"

First Round of Reviewer Comments

Reviewer: 1

Comments to the Author

The authors apply half-broadband PES to investigate electronic vibrational interactions in the compound crescyl violet. Beatmaps that are extracted from 2DES spectra and simulated with a displaced harm. osz. model suggest that for one mode (338 cm-1) a displaced higher excited state is necessary for reproducing the experimental results.

This paper is certainly based on very high-quality experimental and computational data. However, from the beginning it wasn't clear to me if the paper focusses on method development or on the photo-physical properties of crescyl violet. This severely limits the accessibility of the paper. I doubt that a reader who is not already familiar with beatmaps and 2DES will gain much from the explanation on page 6, for example. This is also apparent in the conclusion. The significance of the experimental finding is not really clear. The displacement between S0 and S1 has been reported previously. The involvement of Sn in an ESA experiment is obvious. Displacement of Sn appears to be relevant only for two-photon processes. Such processes are rarely important in photochemistry. On the other hand, the advantages of HB2DES and comparison with other methods are briefly mentioned but not discussed in detail. The authors should focus either on the method or on the photo-physics in CV.

While the line of reasoning is convincing, it would help to discuss and connect the simulation more closely to the experiment. For example, the authors could discuss how the simulation would differ if only 2 PES are considered or if the displacement is different. On page 9, first sentence, the authors claim that excellent agreement between measurement and simulation is achieved. They should present data that supports this claim. Figs. 3 are not sufficient, especially considering the peak split in the experimental data that is explained by anharmonicity but not reproduced in the simulation. It is not clear how the numerical values for the couplings were determined. Was the model fitted to the experimental data? It is also not clear how the authors determined that the displacement between S1 and Sn is similar for both vib. modes. Fig 4 a does not include any

transition from S1 to Sn. In summary, the paper should more convincingly show that 2DES resolves relative excited state displacement.

Additional comments:

SI: The authors should explain why the calculated spectra S3 e and j are vastly different.

Page 6: maybe a scheme like Figure 1 in 10.1021/acs.jpcc.1c09432 would be helpful here to give a brief overview of the method.

Page 8, first paragraph: The authors should help the reader by discussing the resulting beatmaps before moving on to the simulation.

Page 9, second paragraph: This is very dense. Sentences like:

"To prepare an excited state vibrational wavepacket, beating with a positive sign during T, $|e0\rangle\langle e1|$, through a rephasing pathway, the first field-dipole interaction acts on the bra at the energy of the 0-0 electronic transition plus (at least) one quantum of vibrational energy (Figure 4b).",

make it hard to follow the discussion for readers who are not already expert in this field.

Reviewer: 2

Comments to the Author

This is a very interesting manuscript that describes a novel approach for extracting the relative displacements of electronic potential energy surfaces from 2DES measurements. The approach is demonstrated for the S1 and Sn states along two Raman-active vibrational coordinates for a well-studied model system of cresyl violet (CV). The experimental results are consistent with various earlier measurements for CV but the real novelty lies in the analysis of the experimental and simulated coherence beatmaps for the 338 and 585 cm-1 vibrations on S1.

The manuscript is necessarily concise in order to be appropriate for JPCL, which leaves a few points that are not described in enough detail to be accessible for non-experts. However the overall quality of the work is very high and the manuscript provides an insightful new perspective in an area that is

currently very active. The manuscript will be suitable for publication after the authors address the following points.

1) The authors need to better explain how the magnitudes of the Sn displacements Δ(fg) were obtained. They only say that the reported values give "excellent agreement" between experimental and simulated spectra without providing any details. In particular, if the values were determined from trial and error or fitting it would be helpful to know how sensitively the simulated spectra depend on the actual values of the displacements. How the values of the S1 displacements Δ(eg) were obtained needs to be stated more clearly also. The manuscript implies that these may have been taken from reference 21, but it needs to be stated explicitly if that's the case.

2) It's unclear why the line shapes in the simulated beatmaps in Fig 3 are not centered on the markers that indicate the individual Liousville pathways. Are the simulated features simply due to the sum of pathways, including their broadening terms, or is there another reason for the offsets? Similarly, the experimental beatmaps in Fig 3b-3d have much narrower bandwidth in nu3 compared with the simulated spectra, which was not discussed in the text.

3) I'm also unclear if the "most striking discrepancy" described on page 10 (line 20) refers to the presence of a second feature in nu1 or nu3. I think the authors mean nu1, but this follows a paragraph about the dependence on the detection frequency. In either case it needs more explanation. If it's the double feature in nu1 then why can't this be explained by the contributions from first and second overtones of the vibration, as implied by the light dashed lines in the figure? And in any event, what is the explanation of the second feature in the nu3 (which is more evident in 3c and 3d)?

4) The beatmaps for non-rephasing pathways in the SI weren't described in any level of detail. The authors should comment on whether or not there is any meaningful information there that can't be obtained from the rephasing data.

Author's Response to Peer Review Comments:

Please see attached file (which contains a figure and coloured text). We have also submitted a highlighted version of the manuscript to show edits.

We are grateful to both reviewers for their supportive comments and helpful suggestions. Please also excuse the long delay in resubmission. Both reviewers required a better description of the data fitting. In preparing this we decided to improve the ‘resolution’ of the displacement by additional impulsive calculations (see revised text) and then a final full calculation with the pump field. This (especially the latter) took some time. The process and data are described fully in the SI and referenced in the main text (see also below).

In the following the reviewer comments are reproduced in full (black). Our responses are in red and new text is shown in blue. We believe all comments have been addressed.

Reviewer(s)' Comments to Author:

Reviewer: 1

Recommendation: This paper may be publishable, but major revision is needed; I would like to be invited to review any future revision.

Comments:
The authors apply half-broadband PES to investigate electronic vibrational interactions in the compound cresyl violet. Beatmaps that are extracted from 2DES spectra and simulated with a displaced harm. osz. model suggest that for one mode (338 cm-1) a displaced higher excited state is necessary for reproducing the experimental results.

This paper is certainly based on very high-quality experimental and computational data. However, from the beginning it wasn’t clear to me if the paper focusses on method development or on the photo-physical properties of cresyl violet. This severely limits the accessibility of the paper.

It is a little of both. Of course CV is a model system and the S0-S1 transition is well studied by 2DES. Our claim is that by extending the measurements to ESA we observe new coherent dynamics. We then show that we can model these in a way that allows us to access displacements along coordinates in higher excited state. Our claim is that this will be important in understanding and exploiting some of the new photophysical phenomena being observed in higher excited state. This was somewhat lost in the second introductory paragraph:

We performed HB2DES on the system cresyl violet perchlorate (CV) in ethanol (EtOH). CV, a cationic oxazine dye, has been widely studied by one-colour 2DES, leading to a wealth of data on population and coherence dynamics associated with the ground-state bleach (GSB) and stimulated emission (SE).26–30 Utilising the extended range of WLC probe in HB2DES enables access to higher electronic states, Sn, via excited state absorption (ESA), revealing the previously inaccessible coherent dynamics. This allows us to probe displacement among higher excited states through the relative amplitude of wavepackets arising from multiple Raman active modes coupled to different electronic transitions.

Which has been rewritten as follows:

We performed HB2DES on the system cresyl violet perchlorate (CV) in ethanol (EtOH). CV is a

cationic oxazine dye, that has been widely studied by one-colour 2DES, leading to a wealth of data on population and coherence dynamics associated with the ground-state bleach (GSB) and stimulated emission (SE) transitions.26–30 By addressing dynamics in this model system we show that the extended range of the WLC probe in HB2DES enables access to higher electronic states, Sn, via excited state absorption (ESA), revealing coherent dynamics that are inaccessible in most one-colour experiments. We further show that detailed analysis of these ESA measurements allows recovery of displacements among higher excited states, through the relative amplitude of wavepackets arising from multiple Raman active modes coupled to different electronic transitions. The ability to initiate coherent dynamics and probe the structure of higher lying excited states is becoming increasingly important in ultrafast photochemistry. Several recent experiments show that excitation of electronic states higher than S1 leads to novel photophysical and photochemical processes not accessible by other pathways, encompassing processes such as novel pathways in photochromics, charge injection for photovoltaics and anti-Kasha emission.31–35 The present data suggest coherence spectroscopies provide new information and potentially suggest routes to control such phenomena.

I doubt that a reader who is not already familiar with beatmaps and 2DES will gain much from the explanation on page 6, for example.

We added a scheme to aid the understanding and discussion of the beatmap calculation procedure to the SI (Figure S3). This scheme is referred to in page 6. New text reads as:

A schematic illustration of this procedure is shown in the supplementary information, Figure S3.

The scheme and caption are also reported here:

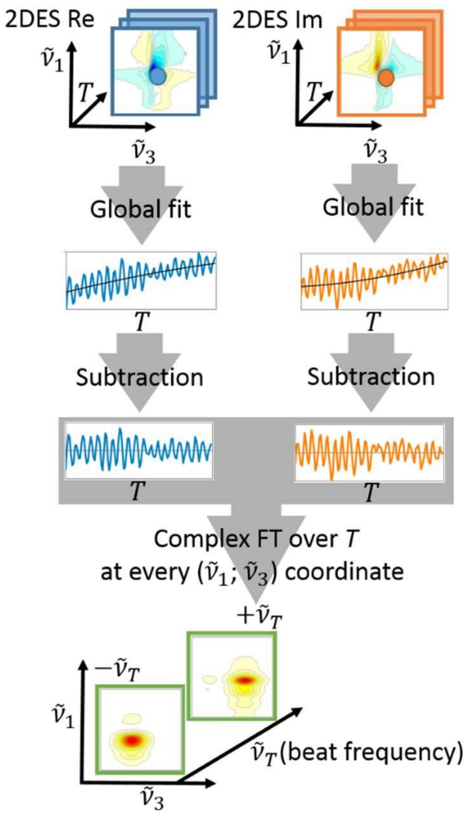

Figure S3 Scheme of the method used to recover positive and negative beatmaps from a complex-valued 2DES rephasing or nonrephasing dataset acquired in even T steps (satisfying the Nyquist sampling criterion). The Re and Im parts of the (rephasing or nonrephasing) signal are independently fit to a sum of exponential decays plus an offset to capture the "slow" population relaxation dynamics. The global fit is then subtracted from the data to isolate the residuals, corresponding to the oscillatory part of the third-order signal. The real and imaginary residuals are summed as Re + iIm to yield a complex-valued matrix which is Fourier transformed over T to obtain a 3D spectrum as a function of \nu_1, \nu_T and \nu_3, where \nu_T is the conjugate variable of the waiting time T. Beatmaps at a specific ±\nu_T are then obtained by slicing the 3D spectrum at that frequency. The beatmaps obtained by this method are shown in Figures 3 and S6.

This is also apparent in the conclusion. The significance of the experimental finding is not really clear. The displacement between S0 and S1 has been reported previously. The involvement of Sn in an ESA experiment is obvious. Displacement of Sn appears to be relevant only for two-photon processes. Such processes are rarely important in photochemistry.

We have now included in the introductory paragraph (above) mention of new processes that can be studied and potentially controlled by understanding dynamics and displacements in higher excited states. As now indicated, we would question 'rarely important' in favour of 'increasingly important'. While undoubtedly still a minority concern the number of reports of new phenomena arising from higher excited states is increasing exponentially.

On the other hand, the advantages of HB2DES and comparison with other methods are briefly mentioned but not discussed in detail. The authors should focus either on the method or on the photo-physics in CV.

We have tried to better highlight why we feel the comparison with other methods (e.g. FSRS) may be important by extending the final paragraph. Both methods can give the displacements, but observation of coherences in HB2DES in ESA requires wavepacket generation in the 'pump' step. Thus, observation of wave packets in the 'product' state is a requirement for any (third order) 'coherent control' scheme.

Finally, it is worth noting the difference between the HB2DES technique and other methods of monitoring displacements between excited states, such as time or frequency domain stimulated Raman scattering (ISRS, FSRS) spectroscopies. Differently from formally six-wave mixing, (χ(5)) experiments such as ISRS, FSRS41–44 and t-2DES,45 in which the actinic pulse is not involved in the preparation of (ground and) excited state nuclear wavepackets, coherent modulations of the Sn←S1 ESA detected in 2DES arise from wavepacket dynamics initiated by the pump pair. In other words, in HB2DES a null displacement along a given coordinate between S1←S0 suppresses wavepacket dynamics in both the GSB+SE and ESA regions, whilst a S1 vibration can be coherently excited in six-wave mixing experiments regardless of its S1←S0 displacement. Thus, the observation of coherent dynamics in product states by HB2DES points directly to wavepacket generation occurring from the ground state, in the pump step.

Is replaced with:

Finally, it is worth noting the difference between HB2DES and other methods of monitoring displacements between excited state vibrational modes, such as stimulated Raman scattering in the time or frequency domain, and transient 2DES spectroscopies.46–50 The vibrational spectra provided by these methods could also be analysed in terms of displacements among higher excited states. Differently from these formally six-wave mixing, (χ(5)) experiments, in which the initial actinic pulse is not involved in the preparation of ground and excited state nuclear wavepackets, coherent modulation of the Sn←S1 ESA

detected in the χ(3) 2DES must arise from wavepacket dynamics initiated by the pump pair. Thus, in HB2DES a negligible displacement along a coordinate between S1⟵S0 will suppress wavepacket dynamics in both the GSB+SE and ESA regions. Conversely, the observation of coherent dynamics in higher excited (or photochemical product) states by HB2DES directly implicates wavepacket generation initiated in the pump step. Consequently, the observation of coherences in ESA by HB2DES can indicate at least the possibility of coherent control of the photochemical processes in higher excited states.

While the line of reasoning is convincing, it would help to discuss and connect the simulation more closely to the experiment. For example, the authors could discuss how the simulation would differ if only 2 PES are considered or if the displacement is different.

With 2 PES the ESA feature could not be reproduced, beatmaps would only report on GSB and SE vibrational coherences. Obviously, no relative displacements between S0-S1 and S1Sn could be obtained from a two level system.

Different displacement values translate to different ratios between the SE and ESA beatmap amplitudes, as shown in Figures S4 and S5, where such ratios are plotted against displacement for rephasing and nonrephasing modelled HB2DES of CV. These values were determined by the less computationally demanding impulsive method, as described below.

On page 9, first sentence, the authors claim that excellent agreement between measurement and simulation is achieved. They should present data that supports this claim. Figs. 3 are not sufficient, especially considering the peak split in the experimental data that is explained by anharmonicity but not reproduced in the simulation. It is not clear how the numerical values for the couplings were determined. Was the model fitted to the experimental data?

The new paragraph describing the model in the manuscript, page 9, is reported here:

The S1-Sn displacements were determined by first calculating 2D spectra using the impulsive method described in Green et al.43 for a range of Sn⟵S1 displacements of the 338 and 585 cm-1 modes. Rephasing and nonrephasing beatmaps were obtained by applying the procedure outlined in Figure S3 to the calculated 2DES datasets. The amplitude ratio between the ESA and the GSB+SE signals of each beatmap was plotted against the displacement along its normal mode coordinate to yield the data in Figures S4 and S5, and compared to the experimental ESA / GSB+SE amplitude ratio, determined by Δ , and shown as a horizontal red line in S4, 5. The best match between the experimental data and impulsive calculation then determined the displacements used with the computationally intense HEOM-PMA method for finite fields, obtaining the HB2DES rephasing (Figure 3) and nonrephasing (Figure S6) beatmaps. Further details on the method are given in the supplementary information.

A more detailed description of the method is given in the SI and reported here:

For a fixed S1-S0 displacement, increasing the Sn-S1 displacement increases the intensity of the ESA coherences relative to the GSB+SE coherences in the vibrational beatmaps. The Sn-S1 displacements, Δ , are therefore obtained by fitting the ratio of the maximum intensity of the ESA coherences ($\upsilon \geq$ 18800 cm^{-1}) vs. the maximum intensity of the GSB+SE coherences ($\upsilon <$ 18800 cm^{-1}) for the calculated beatmaps to the measured value. The values of this ratio for the rephasing ±338 cm^{-1} and ±585 cm^{-1} beatmaps in figure 3 are shown by the red lines in figure S4. The increase in this ratio for increasing Δ

is shown by a series of beatmaps obtained from spectra calculated using the impulsive method described in the appendix of ref. 1 which were used initially to identify an appropriate range for Δ .
Subsequent more intensive calculations using the equation of motion-phase matching approach for finite pulses discussed below then identified Δ =0.08 as the best fit of this ratio for both the 338 and 585 cm^{-1} modes. Figure S5 shows the equivalent for the nonrephasing beatmaps which also supports the fit of Δ =0.08 for both the 338 and 585 cm^{-1} modes.

It is also not clear how the authors determined that the displacement between S1 and Sn is similar for both vib. modes. Fig 4 a does not include any transition from S1 to Sn. In summary, the paper should more convincingly show that 2DES resolves relative excited state displacement.

Figure 4 a shows a SE vibrational coherence diagram, whose amplitude entirely depends on the S0-S1 displacement. The S1-Sn displacement along the 585 mode was determined by evaluating the amplitude of the 585 beatmaps in the ESA region, whose PES diagram is not shown in figure 4, but the relevant DSFD is reported in the SI. The new data support this assignment.

Additional comments:

SI: The authors should explain why the calculated spectra S3 e and j are vastly different.

We agree there is an unexpected difference in the intensities of the experimental and calculated nonrephasing -585 cm-1 beatmaps. Whilst both figure S3(e) and (j) have an intense SE peak at υ = 16000 cm-1, the equally intense GSB peak at υ = 17000 cm-1 in the calculated spectrum, S3(j), is still visible in the experimental beatmap, S3(e), but significantly weaker. Similarly the ESA peak is visible, but much weaker in (j) compared with (e). As the SE and GSB peaks appear at the same coordinates in both (e) and (j) there is a good agreement between the experiment and model, though clearly the observed enhancement of the SE is absent from the model and we continue to explore its origins.

This difference is now explicitly noted in the manuscript, page 9:

Excellent agreement between experimental and calculated 2D rephasing beatmaps of CV is achieved with displacements between the ground and higher excited states of Δ = 0.71 for the 585 cm^{-1} mode and Δ = 0.26 for 338 cm^{-1} mode. The same values yielded reasonable agreement for the nonrephasing data (shown in Figure S6) except for the lineshape of the -585 cm-1 beatmap, which shows an enhancement of the SE peak which is absent from the model.

And discussed in the SI:

The calculated beatmaps reproduce the much greater ESA intensity for the 338 cm^{-1} mode, but the ESA peak in the calculated -585 cm^{-1} beatmap in fig. S6(j) is more intense than in the experimental beatmap. This is a consequence of the enhancement of the SE peak at υ = 16000 cm^{-1} in fig S6(e) compared with the peak at υ = 17000 cm^{-1} which is absent from the model, where these peaks have the same intensity in fig. S6(j).

Page 6: maybe a scheme like Figure 1 in 10.1021/acs.jpcc.1c09432 would be helpful here to give a brief overview of the method.

We added a scheme to aid the discussion of the beatmap extraction procedure to the SI as stated earlier.

Page 8, first paragraph: The authors should help the reader by discussing the resulting beatmaps before moving on to the simulation.

Now the beatmaps are discussed in terms of peak positions and linewidths before comparing them with the calculations, new text on page 8 reads as:

All the experimental beatmaps present intense signals around the GSB+SE maximum (ν̃_3 = 16000 cm-1). A medium intensity signal is detected around the ESA maximum (ν̃_3= 19400 cm-1) for the -338 cm-1 beatmaps, whilst very weak signals in the ESA region are present in the +338 cm-1 and ±585 cm-1 beatmaps. These signals are due to vibrational coherences active in either S0 or S1, and their dependence on the (ν̃_1;ν̃_3) coordinates can be explained in terms of the displaced harmonic oscillator (DHO) model. This model predicts how a vibrational mode coupled to an electronic transition gives rise to wavepacket oscillations during T and thus beatmap amplitude at specific excitation and detection frequency coordinates. These coordinates are predicted by double-sided Feynman diagrams (DSFDs) and marked by coded symbols superimposed on the calculated beatmaps in Figure 3g-j.

Page 9, second paragraph: This is very dense. Sentences like:
"To prepare an excited state vibrational wavepacket, beating with a positive sign during T, $|e0\rangle\langle e1|$, through a rephasing pathway, the first field-dipole interaction acts on the bra at the energy of the 0-0 electronic transition plus (at least) one quantum of vibrational energy (Figure 4b).", make it hard to follow the discussion for readers who are not already expert in this field.

We agree that this sentence is hard to follow, we modified the discussion and the new text reads as:

The phase-matching conditions of 2DES cause the beatmap signals due to rephasing +338/+585 cm-1 excited state vibrational coherences to appear blue-shifted, along the excitation axis, by at least one quantum of vibrational energy from the 0-0 electronic transition frequency at 16250 cm-1. This effect is illustrated by the DSFD in Figure 4b. The same phase-matching argument causes the rephasing -338/-585 cm-1 beatmaps to be centred at the 0-0 electronic transition frequency (16250 cm-1), as shown by the DSFD in Figures 4d, S6 and S8.

Additional Questions:
Urgency: Moderate

Significance: High

Novelty: Top 10%

Scholarly Presentation: Moderate

Is the paper likely to interest a substantial number of physical chemists, not just specialists working in the authors' area of research?: No

Reviewer: 2

Recommendation: This paper is publishable subject to minor revisions noted. Further review is not needed.

Comments:
This is a very interesting manuscript that describes a novel approach for extracting the relative displacements of electronic potential energy surfaces from 2DES measurements. The approach is demonstrated for the S1 and Sn states along two Raman-active vibrational coordinates for a well-studied model system of cresyl violet (CV). The experimental results are consistent with various earlier measurements for CV but the real novelty lies in the analysis of the experimental and simulated coherence beatmaps for the 338 and 585 cm-1 vibrations on S1.

The manuscript is necessarily concise in order to be appropriate for JPCL, which leaves a few points that are not described in enough detail to be accessible for non-experts. However the overall quality of the work is very high and the manuscript provides an insightful new perspective in an area that is currently very active. The manuscript will be suitable for publication after the authors address the following points.

1) The authors need to better explain how the magnitudes of the Sn displacements Δ(fg) were obtained. They only say that the reported values give "excellent agreement" between experimental and simulated spectra without providing any details. In particular, if the values were determined from trial and error or fitting it would be helpful to know how sensitively the simulated spectra depend on the actual values of the displacements. How the values of the S1 displacements Δ(eg) were obtained needs to be stated more clearly also. The manuscript implies that these may have been taken from reference 21, but it needs to be stated explicitly if that's the case.

Reviewer 1 also raised the question of how the data were fitted to the model. We added an explanation on how the data were analysed and relative displacements were obtained, including some extra impulsive calculations as discussed above (see also p9 of text)

Regarding the S0-S1 displacements, the new text, page 9 now explicitly says that these were taken from reference 21 as they well reproduced our experimental observations. To highlight this new we also added (page 9) one sentence, reproduced here:

We thus used these literature values in our calculations.

2) It's unclear why the line shapes in the simulated beatmaps in Fig 3 are not centred on the markers that indicate the individual Liouville pathways. Are the simulated features simply due to the sum of pathways, including their broadening terms, or is there another reason for the offsets? Similarly, the experimental beatmaps in Fig 3b-3d have much narrower bandwidth in nu3 compared with the simulated spectra, which was not discussed in the text.

As suggested, the “offsets” from the markers arise from the overlapping finite linewidths. New text was added to explain the effect on the beatmap features (page 8):

Due to experimental linewidths in excess of a few hundred wavenumbers, signals arising from multiple DSFDs merge into broad features located between the point amplitudes predicted by individual DSFDs, rather than being detected as resolved peaks. Such an effect is especially relevant for beatmaps of low frequency vibrations such as the 338 cm-1 mode.

Regarding the difference between experimental and calculated beatmap bandwidths, it arises from how broadening is introduced in the model. The broadening, related to the dephasing time, is chosen to yield good agreement between the simulated and measured absorptive 2DES bandwidths. Although, as in the model a single vibration accounts for the broadening which is, in reality, due to a multitude of modes, this leads to overestimation of its dephasing time, translating to pronounced broadening in the beatmaps. The explanation of such effect was added to the manuscript, page 8, and reads as:

Finally, the ν bandwidth differences between experimental (narrow) and calculated (broad) beatmaps can be rationalised on the basis of how the calculated absorptive 2DES is broadened to yield good agreement with the experimental linewidth. In the model, a single vibration accounts for the entirety of the experimentally observed broadening of the electronic transition. Such overestimation of the contribution of a single vibration translates to greater broadening in the calculated beatmaps.

3) I'm also unclear if the "most striking discrepancy" described on page 10 (line 20) refers to the presence of a second feature in nu1 or nu3. I think the authors mean nu1, but this follows a paragraph about the dependence on the detection frequency. In either case it needs more explanation. If it's the double feature in nu1 then why this can't be explained by the contributions from first and second overtones of the vibration, as implied by the light dashed lines in the figure? And in any event, what is the explanation of the second feature in the nu3 (which is more evident in 3c and 3d)?

The discrepancy refers to the elongation on the excitation axis. This is now explicitly mentioned, page 10.

The most striking discrepancy between measured and calculated beatmaps is the double peak structure elongated along ṽ_1

We agree with the reviewer that the elongation along the excitation axis of the +338 cm-1 beatmap in the GSB/SE region could arise from pathways in which the first interaction with the pump pulse excites up to three quanta of this mode, which are within its bandwidth. This signal cannot be reproduced by the calculations because of the narrower Gaussian pump spectrum used for the calculations. Following this suggestion we added horizontal dashed lines at 0-0 electronic + 3 x 338 cm-1 to Figure 3 b,c and text (page 11) that reads as:

Another possible explanation of these features is the presence of pathways involving excitation up to three quanta of the 338 mode, covered by the experimental pump spectrum (see horizontal dashed lines) but not present in the spectra calculated with a narrower Gaussian pump pulse spectrum. However, as a single-mode DHO would not reproduce the feature centred at ṽ_3=15550 cm-1 in Figure 3c or the pattern observed below diagonal in the nonrephasing negative data (Figure S6c) these unpredicted features are most likely due to coupling between low frequency Raman active vibrations

Conversely, the feature appearing at nu3 = 15700 cm-1 in the rephasing +585 cm-1 beatmap (Figure 3d) is consistent with the DHO model (see green squares in Figure 3i). The fact that these appear as two resolved peaks in the experimental whilst the modelled beatmap only shows one broad feature relates to the

broadening argument discussed above (the dephasing of the calculated spectra was overestimated to broaden the simulated SS absorption).

4) The beatmaps for non-rephasing pathways in the SI weren't described in any level of detail. The authors should comment on whether or not there is any meaningful information there that can't be obtained from the rephasing data.

We thank the reviewer for pointing this out. The nonrephasing beatmaps are indeed containing the same information of the rephasing data shown in the manuscript. Although, their consistency with rephasing data is further proof of the soundness of the experimental (modelled) data and of the beatmap extraction method. A description of the nonrephasing data was added to the SI and reproduced here:

The nonrephasing beatmaps in fig. S6 show the same features as the rephasing beatmaps in figure 3. The experimental beatmaps all have peaks in the GSB+SE region which are significantly more intense than peaks due to ESA coherences. However, the much greater intensity of ESA coherences in the ±338 cm^{-1} beatmaps compared to the ±585 cm^{-1} beatmaps again indicates a much greater relative displacement for the 338 cm^{-1} mode than for the 585 cm^{-1} mode. The calculated beatmaps show good agreement with the experimental data, with peaks aligned with respect to the excitation axis, υ , although limited in this axis by the slightly narrower Gaussian pump spectrum. The calculated beatmaps reproduce the much greater ESA intensity for the 338 cm^{-1} mode, but the ESA peak in the calculated -585 cm^{-1} beatmap in fig. S6(j) is more intense than in the experimental beatmap. This is a consequence of the enhancement of the SE peak at υ = 16000 cm^{-1} in fig S6(e) compared with the peak at υ = 17000 cm^{-1} which is absent from the model, where these peaks have the same intensity in fig. S6(j). Similarly, the multiple peaks in the GSB+SE region of the ±338 cm^{-1} experimental beatmaps, as in the rephasing beatmaps, are not observed in the calculated beatmaps as the model accounts for a single vibrational mode only. Thus the model cannot account for coupling with other vibrational modes and overestimates the broadening of this mode in an effort to match the experimental lineshape which is in fact the result of multiple vibronic progressions.

Additional Questions:
Urgency: Top 10%

Significance: Top 10%

Novelty: High

Scholarly Presentation: High

Is the paper likely to interest a substantial number of physical chemists, not just specialists working in the authors' area of research?: Yes
